# Mapping the Intellectual Architecture of Education for Sustainable Development in Higher Education: A Bibliometric and AI-Assisted Thematic Synthesis

**DOI:** 10.12688/f1000research.163621.1

**Published:** 2026-05-06

**Authors:** Segundo Francisco Segura-Altamirano, Julio Santos Hilario-Vargas, Lindon Vela-Melendez, Carmen Graciela Arbulu-Perez-Vargas, Jose Teodoro Reupo-Periche, Jhon Wiston Garcia-Lopez, Mauro Adriel Rios-Villacorta, Hugo Javier Chiclayo-Padilla, Diana Mercedes Castro-Cárdenas

**Affiliations:** 1 Universidad Nacional Pedro Ruiz Gallo Facultad de Ciencias Fisicas y Matematicas, Lambayeque, Lambayeque, 14100, Peru; 2 Universidad Nacional de Trujillo,Av. Juan Pablo II s/n, Trujillo, 13001, Peru; 3 Universidad Cesar Vallejo, Pimentel, Lambayeque, 14110, Peru

**Keywords:** Education for Sustainable Development, Higher Education, Science Mapping, Sustainability Competencies, Green Skills, Artificial Intelligence

## Abstract

**Background:**

Despite the exponential growth of research on Education for Sustainable Development (ESD) in higher education, the field remains characterized by geographic concentration, methodological constraints, and conceptual fragmentation between established competence frameworks and emerging demands such as climate literacy and green skills. No prior review has simultaneously mapped the bibliometric architecture and conducted an AI-assisted inductive thematic synthesis of this domain.

**Methods:**

A dual-method design integrated bibliometric network analysis—co-citation, bibliographic coupling, and keyword co-occurrence—with artificial intelligence-assisted inductive thematic synthesis. A total of 442 peer-reviewed documents were retrieved from Scopus, Web of Science, and ERIC (2015–2026) through systematic search, deduplication, and PICO-based relevance screening. Thematic extraction was performed using two complementary large language model architectures (Qwen3-235B and DeepSeek V3.2), with a stringent hallucination-control verification protocol achieving a 92% alignment score; 37 hallucination flags were subjected to human expert review.

**Results:**

The corpus exhibits exponential growth since 2019, with Spain, the United Kingdom, and Germany accounting for over one-third of output. Five thematic clusters emerged: curriculum integration, transformative pedagogies, institutional and faculty roles, sustainability literacy and behavioral outcomes, and digital innovation. Surveys and case studies dominate research designs (38%), while experimental approaches represent only 4.0% of the corpus. Green skills and climate literacy remain conceptually peripheral despite their policy urgency, institutional collaboration is extremely fragmented (20.3% of institutions isolated), and faculty populations are severely understudied (7.0%) relative to students (32.7%). Fifty-six knowledge gaps were identified, with methods-by-concepts coverage reaching only 64.0%.

**Conclusions:**

The field urgently requires a methodological pivot toward longitudinal and experimental designs, amplification of Global South scholarship, systematic pedagogical theorization of simulation-based approaches, and deliberate cross-institutional consortia to transition from conceptual proliferation to evidence-based pedagogical science.

## Introduction

The growing severity of global socio-ecological challenges—including climate change, biodiversity loss, and social inequalities—has placed sustainable development at the center of twenty-first century policy and educational agendas. In response, the international community has increasingly recognized education as a key driver of the societal changes needed to address these challenges. The United Nations’ Decade of Education for Sustainable Development (2005–2014) sought to integrate the principles and practices of sustainable development into all aspects of education ( Grabovska & Grabowski, 2009; Öztürk, 2017). While this initiative elevated environmental awareness and institutionalized sustainability within global policy discourse, critical reflections suggest that treating sustainable development primarily as a top-down policy objective generated persistent misalignments between rhetorical commitments and the anthropocentric paradigms governing dominant educational systems. Moving beyond these limitations may require reconceptualizing sustainability not as a policy add-on, but as a perspective that reshapes how individuals and institutions relate to the natural world ( Martin et al., 2013; Ontong & Grange, 2015).

Building upon these foundations, the adoption of the 2030 Agenda for Sustainable Development recalibrated the global educational trajectory. Sustainable Development Goal 4 commits to ensuring inclusive, equitable, and high-quality education, while Target 4.7 mandates that all learners acquire the knowledge and skills necessary to promote sustainable development, encompassing human rights, gender equality, and global citizenship ( Alonso et al., 2023; Joseph & Areepattamannil, 2025). Within this framework, Higher Education Institutions play an important role: universities and vocational training centers contribute to the formation of future professionals, policymakers, and educators, and their capacity to embed sustainability into their curricula can influence broader sustainable development efforts ( Aver et al., 2021; Demaidi & Al-Sahili, 2021; Ferk Savec & Jedrinović, 2025). While some institutions have developed models that embed sustainability into their strategic planning ( Oliveira-Melo et al., 2025; Sahin, 2025), many institutions continue to face fragmented implementation and institutional inertia.

Central to this effort is the discourse on sustainability competencies. Foundational frameworks have conceptualized these competencies as interlinked domains encompassing systems thinking, anticipatory competence, normative competence, interpersonal skills, and strategic action capabilities ( Besong & Holland, 2015; Laasch et al., 2023; Mukhtar et al., 2019). These models postulate that students must not only understand the complex, non-linear dynamics of socio-ecological systems but also possess the ethical grounding and collaborative skills required to navigate value conflicts and implement systemic solutions in real-world contexts. However, mapping these theoretical constructs onto tangible, assessable learning outcomes remains an ongoing challenge, as the transdisciplinary nature of sustainability frequently clashes with the compartmentalized structures of university curricula, leaving educators with limited guidance on how to systematically foster and evaluate these attributes across diverse student cohorts.

These competency goals have prompted a re-evaluation of pedagogical methodologies. Traditional transmissive modes of instruction are increasingly seen as insufficient for fostering the learning needed to address complex sustainability problems ( Hedden et al., 2017; Sundman et al., 2025). Interventions such as project-based learning, service-learning, and community-engaged research are documented as effective vehicles for bridging theoretical knowledge and real-world application ( Aigbe et al., 2025; Chien & Chien, 2025; Johan & Turan, 2016; Mangalore et al., 2025). Yet the successful deployment of these innovative pedagogies is contingent upon the professional development of university faculty. Research points to a bottleneck at the level of educator training: while institutions may declare commitments to sustainability, limited professional development opportunities leave many academics without the preparation needed to design and facilitate competence-oriented curricula ( Batorczak & Klimska, 2020; Singer-Brodowski et al., 2026).

As the global environmental crisis accelerates, the conceptual boundaries of education for sustainable development have expanded into specialized sub-domains. Climate literacy has emerged as a distinct yet related area, requiring interdisciplinary understanding of the Earth’s climate system and the capacity to make informed decisions regarding mitigation and adaptation strategies ( Lay, 2016; Limaye et al., 2020). Climate change is increasingly understood as both a socio-economic and public health concern, supporting its integration across disciplines from engineering to health sciences ( Fuertes et al., 2020; García-Vinuesa et al., 2020; Phiri et al., 2025; Szczepankiewicz et al., 2021).

In parallel, macroeconomic factors have brought sustainability education and workforce development closer together, as reflected in the growing discourse on green skills. As nations commit to decarbonization targets and circular economy models, labor markets are undergoing structural transformation, generating demand for professionals equipped to accelerate this transition ( Branca et al., 2022; Costa et al., 2025; Mukhtar et al., 2022; Storonyanska et al., 2025). The development of competence taxonomies signifies policy efforts to standardize green skills; however, empirical studies reveal persistent mismatches between academic curricula and the evolving requirements of sustainable industries ( Ah-Lian et al., 2019; Barna & Csete, 2022; García Hernández et al., 2025; Yang & Omar, 2026).

These compartmentalized responses have led to calls for whole-institution approaches, based on the argument that sustainability cannot be effectively taught if contradicted by institutional practices ( Keryan et al., 2020; Schopp et al., 2020). Transforming campuses into living laboratories can bridge theory and practice ( Al-Nuaimi & Al-Ghamdi, 2022; Oe et al., 2022), yet hierarchical structures, resource constraints, and entrenched academic metrics frequently impede comprehensive transformation ( Alsharif et al., 2020; Fischer et al., 2015; Nogueiro & Saraiva, 2023; Ruiz-Mallén & Heras, 2020).

Simultaneously, digital transformation—including e-learning platforms, gamification, and generative artificial intelligence—offers potential to scale sustainability education ( Ferk Savec & Jedrinović, 2025; Jarillo et al., 2019), while raising concerns about exacerbating inequalities and weakening the affective connections foundational to sustainability competence ( Durrani et al., 2023; Okulich-Kazarin et al., 2024). Beyond these pedagogical challenges, the research field exhibits significant methodological limitations, including over-reliance on self-reported surveys and small-scale case studies, and an acute scarcity of longitudinal or experimental designs ( Dancz et al., 2017; Probst, 2022).

Given the rapid growth, conceptual diversification, and methodological heterogeneity characterizing this literature, there is a need for comprehensive synthesis. This terminological fragmentation—spanning ESD, climate literacy, green skills, and sustainability literacy as partially overlapping yet operationally distinct constructs—necessitates careful search strategy validation to ensure comprehensive yet precise retrieval across databases. As the field expands, it risks epistemic fragmentation, wherein distinct research traditions operate in isolation, generating redundant frameworks and failing to consolidate a cohesive evidence base. While previous reviews have addressed specific regions ( Hallinger et al., 2024), targeted sub-disciplines ( Nakad et al., 2024; Narong & Hallinger, 2024), or specific practices ( Lim et al., 2022), a global mapping that examines the intellectual structure of the broader multidisciplinary field is currently lacking.

In response, the present study proposes a comprehensive bibliometric review and advanced science mapping of the global literature on sustainable development education in higher and vocational contexts. By combining network analysis techniques—including reference co-citation, bibliographic coupling, and keyword co-occurrence—with artificial intelligence-assisted inductive thematic synthesis and systematic knowledge gap detection, this review aims to: (1) trace the temporal dynamics and geographic distribution of research output across 442 documents spanning 2015–2026; (2) elucidate the intellectual architecture of the field through co-citation and coupling networks; (3) interrogate prevailing methodological designs, study populations, and pedagogical interventions; (4) inductively synthesize the thematic core of the literature; and (5) systematically identify structural, declared, and emergent knowledge gaps through coverage matrix analysis to define a prioritized research agenda for the next generation of ESD scholarship in higher education and vocational education contexts. By characterizing these intellectual structures and identifying knowledge gaps, this review aims to provide scholars, curriculum designers, and higher education leaders with an evidence-based overview to inform future research and pedagogical development in the context of the 2030 Agenda.

## Methodology

**Figure 1.**
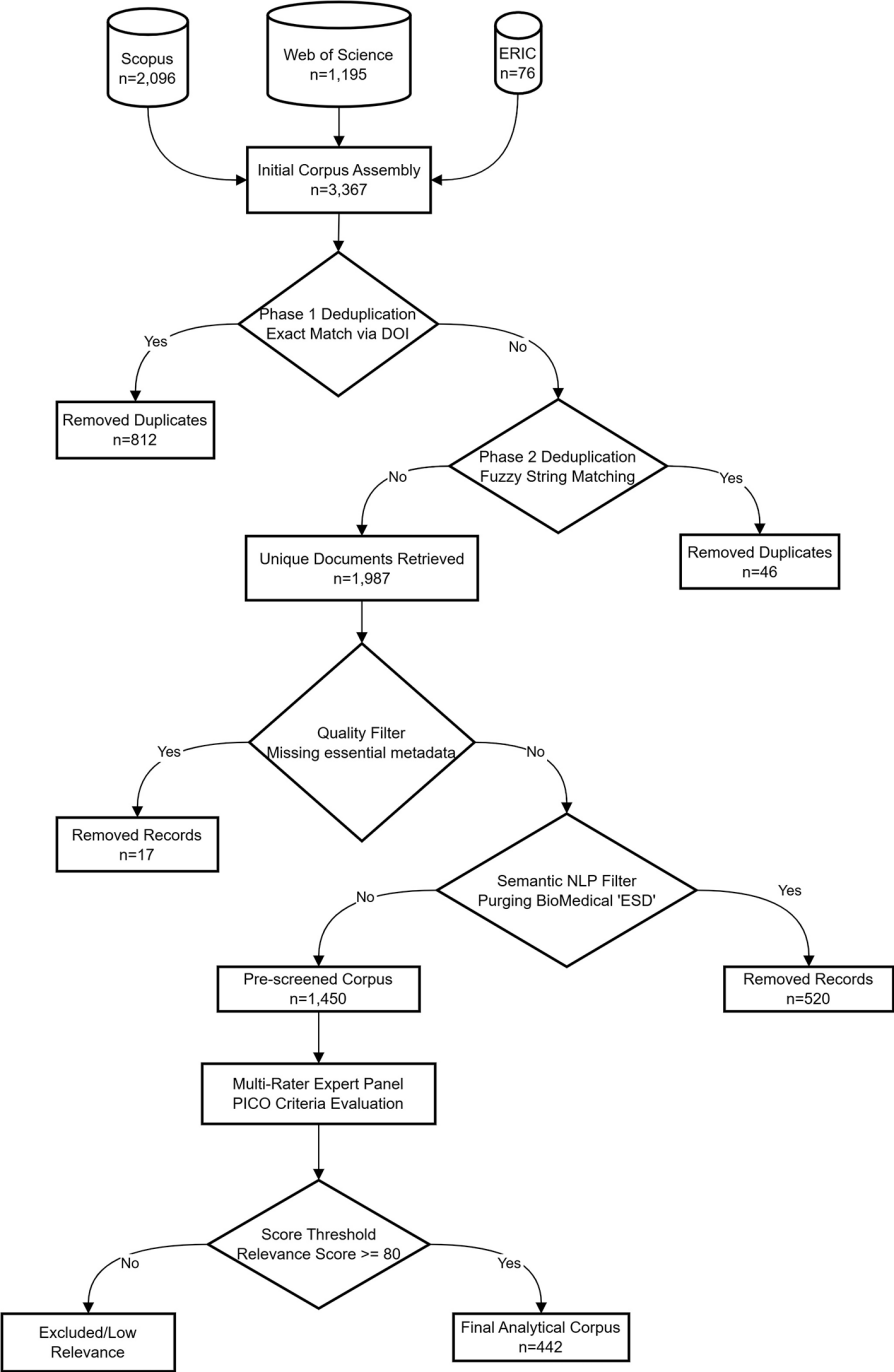
Data acquisition, deduplication, and screening flow. Three-database retrieval from Scopus ( *n* = 2,096), Web of Science ( *n* = 1,195), and ERIC ( *n* = 76) totaling 3,367 raw records, processed through sequential deduplication (DOI exact matching and Levenshtein fuzzy string matching), quality filtering, semantic NLP screening of biomedical homonyms, and PICO-based multi-rater relevance evaluation, yielding the final analytical corpus ( *n* = 442).

This study employed an integrated methodological design combining macroscopic science mapping algorithms with an inductive, artificial intelligence-assisted thematic analysis. The dual approach enables navigating the expansive volume of contemporary literature while preserving the depth required to interpret the complex integration of sustainability within higher education curricula, pedagogical models, and institutional structures. The research framework was structured in five sequential stages: (1) search strategy validation and multi-database adaptation, (2) systematic data acquisition and screening, (3) deduplication, quality filtering, and relevance screening, (4) science mapping and bibliometric network analysis, and (5) inductive thematic clustering with hallucination-controlled verification.  Figure 1 synthesizes the complete data pipeline from retrieval to the final analytical corpus.

### Search Strategy Validation and Multi-Database Adaptation

Prior to the systematic retrieval, the Boolean search strategy was constructed and validated in Scopus through an incremental marginal contribution protocol. Each candidate term was tested for its incremental retrieval yield and potential noise introduction. This iterative process confirmed the inclusion of terms such as *vocational education*/ *VET*, *climate literacy*, green skill*, and *sustainability literacy*, while *curriculum integration* and *carbon literacy* were excluded due to insufficient marginal contribution or excessive retrieval noise. The validated Scopus query adopted a two-domain structure combining the sustainability paradigm with the educational context:

(“education for sustainable development” OR “esd” OR “climate literacy” OR “green skill*” OR “sustainability literacy”) AND (“higher education” OR “university” OR “tertiary education” OR “vocational education” OR “VET”)

Once validated in Scopus, the query was syntactically adapted to the field structures and controlled vocabularies of Web of Science Core Collection and ERIC, preserving the same conceptual logic across all three databases.

### Data Acquisition

The systematic literature retrieval was executed on February 25, 2026, targeting peer-reviewed outputs across Scopus, Web of Science Core Collection, and ERIC. The inclusion of these three platforms integrates the broad multidisciplinary coverage of Scopus and WoS with the specialized pedagogical depth provided by ERIC. The validated Boolean strategy was applied to titles, abstracts, and author keywords across all three databases. Only peer-reviewed journal articles, conference proceedings, and book chapters were retained; editorials and systematic reviews were excluded to prevent epistemological circularity. The temporal scope was deliberately unconstrained, extending up to the date of retrieval.

### Data Processing, Deduplication, and Relevance Screening

The initial retrieval yielded 3,367 raw records (Scopus: 2,096; WoS: 1,195; ERIC: 76) ( Diez-Junguitu & Peña-Cerezo, 2026). Metadata from discrete sources were merged into a unified dataset using customized Python scripts. Deduplication proceeded sequentially: exact DOI matching identified 812 duplicates, followed by fuzzy string matching on titles using the Levenshtein distance algorithm, which detected an additional 46, reducing the corpus to 1,987 unique documents. The overlap analysis revealed significant Scopus–WoS convergence (829 shared records), while ERIC contributed predominantly unique pedagogical records, validating its inclusion.

Subsequently, records lacking essential metadata were eliminated ( *n* = 17), and a natural language processing filter purged 520 biomedical entries erroneously indexed under the polysemic acronym “ESD” (Endoscopic Submucosal Dissection), yielding a pre-screened corpus of 1,450 documents.

A final relevance screening was conducted through a multi-rater expert panel comprising three reviewers with differentiated methodological and thematic roles: a lead methodologist, an ESD and sustainability education expert, and a climate literacy and green skills specialist. Each reviewer independently evaluated every document against PICO-adapted criteria: Population—higher education or vocational learners and educators; Intervention—ESD-related pedagogical, curricular, or institutional actions; Comparison—accepted flexibly to include non-experimental internal benchmarks, not restricted to controlled designs; and Outcome—sustainability competencies, literacy, behavioral change, or institutional transformation. Each reviewer issued a categorical decision (include, exclude, or uncertain) accompanied by a confidence rating and dimension-by-dimension justification. A composite relevance score was computed from the averaged confidence ratings weighted by the categorical decisions; only documents achieving a threshold of $$80 were retained. Discrepant cases were resolved through structured majority agreement. The retained corpus of 442 documents exhibited 98.4% unanimous agreement across all three reviewers and 100% majority agreement, confirming the stability and reliability of the selection threshold (100% abstract and title availability; 92.9% DOI completeness).

### Science Mapping and Bibliometric Analysis

The macroscopic architecture of the corpus was evaluated through science mapping methodologies designed to elucidate both the social networks of knowledge production and the latent cognitive structures governing the discipline ( Mazov et al., 2020; Wang et al., 2020). Descriptive statistics were first generated to map publication trajectories, annual growth rates, and the most prolific actors—authors, institutions, and countries. Relational analyses then employed keyword co-occurrence to identify underlying epistemic themes ( Callon et al., 1983; Torcătoru et al., 2022), bibliographic coupling to measure document similarity through shared references and detect invisible colleges ( Karimi et al., 2025; Kessler, 1963), and reference co-citation to uncover the intellectual pillars of the field ( Ali et al., 2019; Yildiz, 2019). Network visualizations were generated using VOSviewer, with temporal overlays permitting the differentiation between consolidated conceptual areas and nascent research frontiers. A state-of-the-art knowledge gap analysis further examined methods-by-concepts coverage matrices to systematically identify structural, declared, and emergent gaps across the corpus.

### Artificial Intelligence-Assisted Inductive Thematic Analysis

To transcend the macroscopic limitations of network analysis and capture the qualitative depth embedded within the substantive text, this review incorporated a Large Language Model-assisted inductive thematic analysis ( Moia et al., 2026; Wen et al., 2026). The 442 abstracts were processed through two complementary architectures: Qwen3-235B for thematic extraction and DeepSeek V3.2 for cross-thematic reasoning. The use of generative AI in qualitative synthesis is an emerging approach that can increase analytical capacity, though it requires careful methodological oversight ( López-Pineda et al., 2025; Ramírez-Correa et al., 2026).

**Figure 2.**
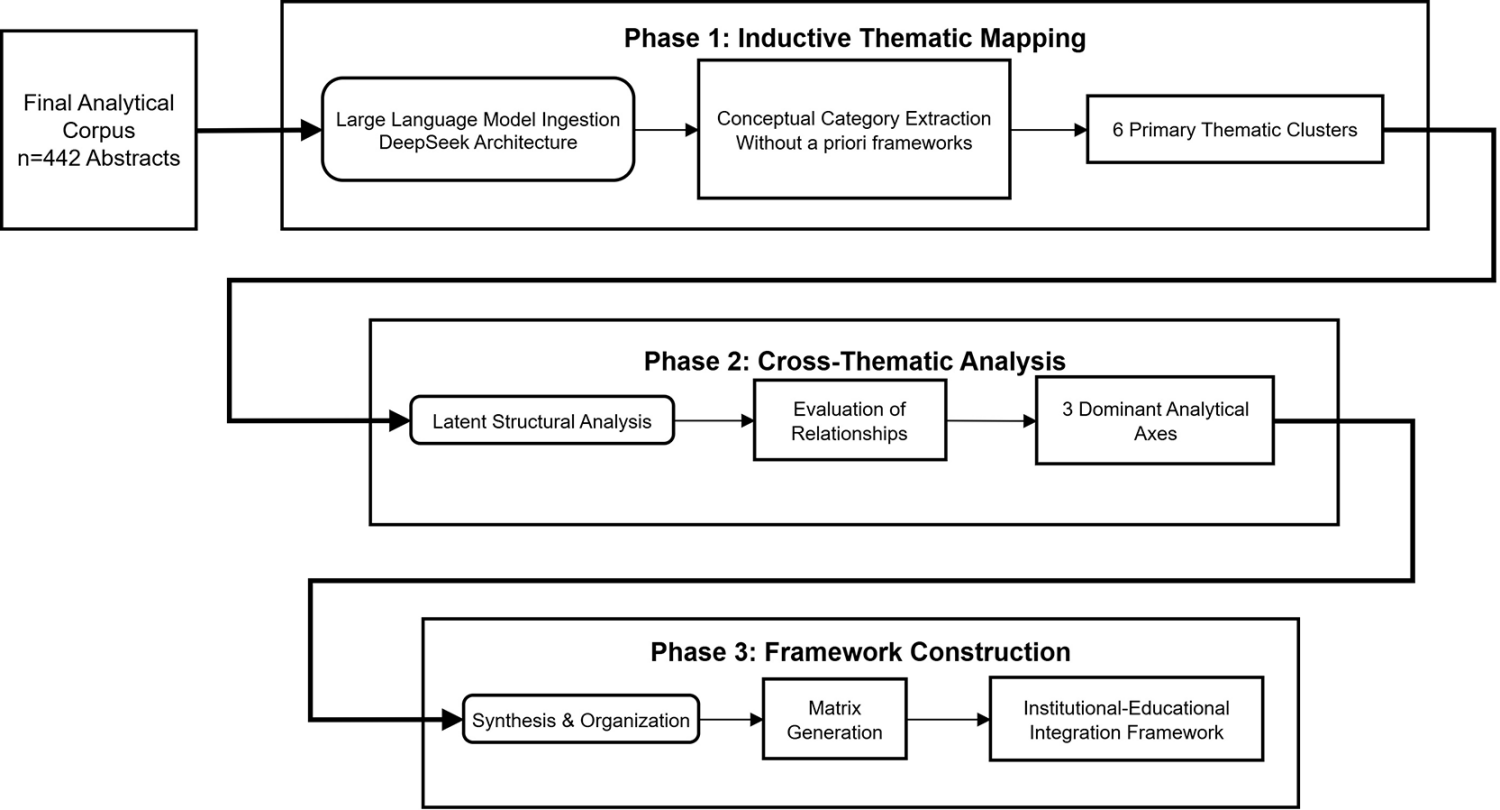
Three-phase inductive thematic analysis pipeline. Phase 1 (Inductive Thematic Mapping): Large Language Model ingestion of 442 abstracts across 15 analytic batches and emergent category extraction yielding five thematic clusters. Phase 2 (Cross-Thematic Analysis): latent structural analysis identifying dominant analytical axes and cross-thematic relationships. Phase 3 (Framework Construction): integrative framework linking institutional, pedagogical, and outcome dimensions.

The analysis proceeded through a three-phase inductive pipeline (  Figure 2). In Phase 1 (Inductive Thematic Mapping), the corpus was processed in 15 analytic batches to identify and define emergent conceptual categories without the imposition of *a priori* theoretical frameworks. This iterative process yielded five primary thematic clusters characterizing the current discourse. In Phase 2 (Cross-Thematic Analysis), the system analyzed the latent structural relationships binding these themes, surfacing dominant analytical axes that delineate the field’s organizing tensions. In Phase 3 (Framework Construction), the synthesized axes were utilized to construct integrative frameworks linking institutional, pedagogical, and outcome dimensions.

**Figure 3.**
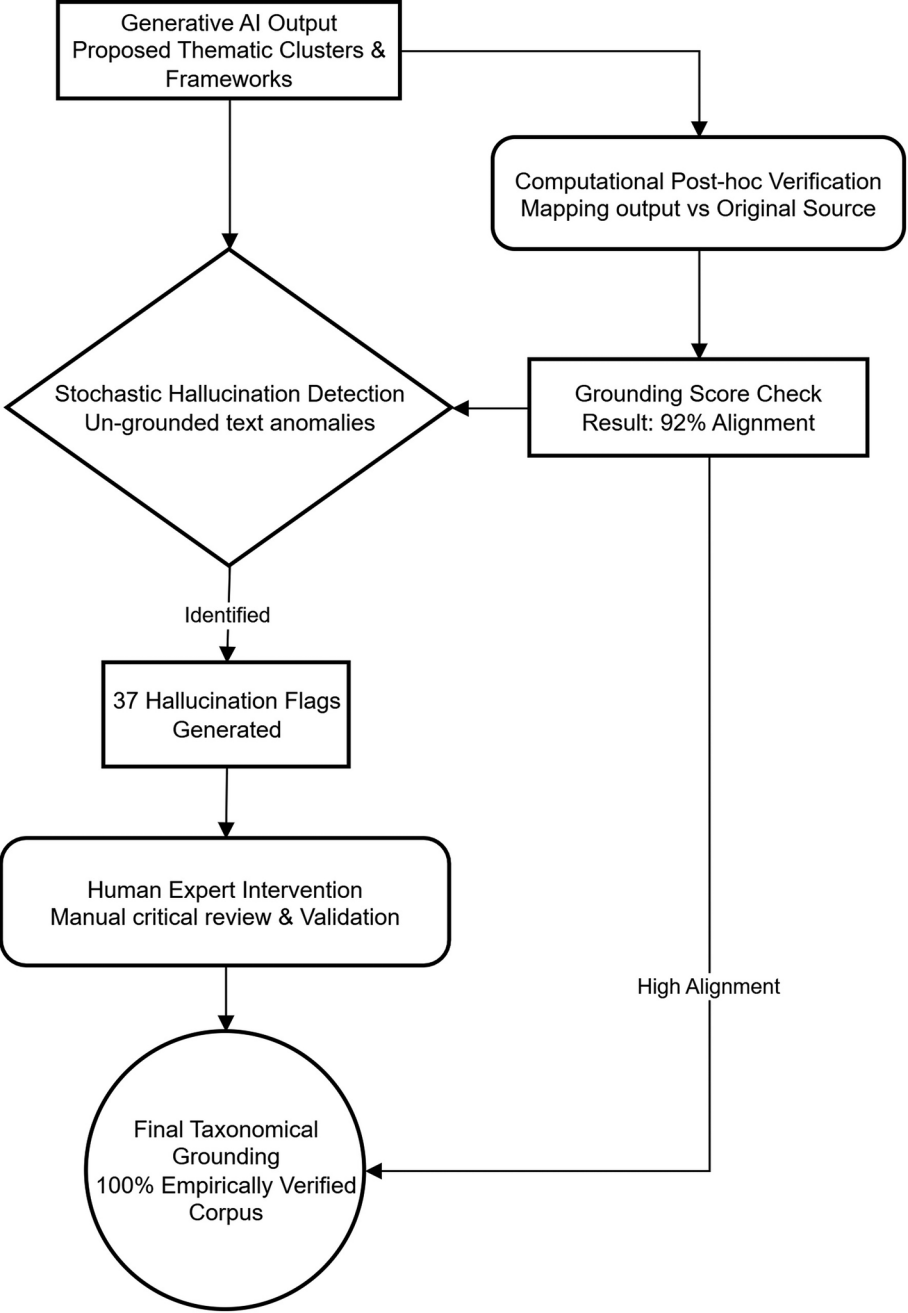
Hallucination control and verification protocol. Computational post-hoc verification of AI-generated thematic outputs against original source abstracts. The protocol yielded a 92% alignment score; 37 hallucination flags were identified and submitted to human expert review for manual validation or correction.

To rigorously mitigate the inherent risks of stochastic algorithmic hallucination ( Luna Chontal et al., 2026), a stringent verification protocol was embedded into the pipeline (  Figure 3). The platform computationally verified thematic propositions directly against the original source text of the abstracts, yielding an alignment score of 92%. A total of 37 hallucination flags were identified and manually reviewed; all instances were critically validated or corrected by the research team to ensure the fidelity of the qualitative synthesis.

## Results

### Descriptive Profile and Temporal Dynamics of the Corpus

**Figure 4.**
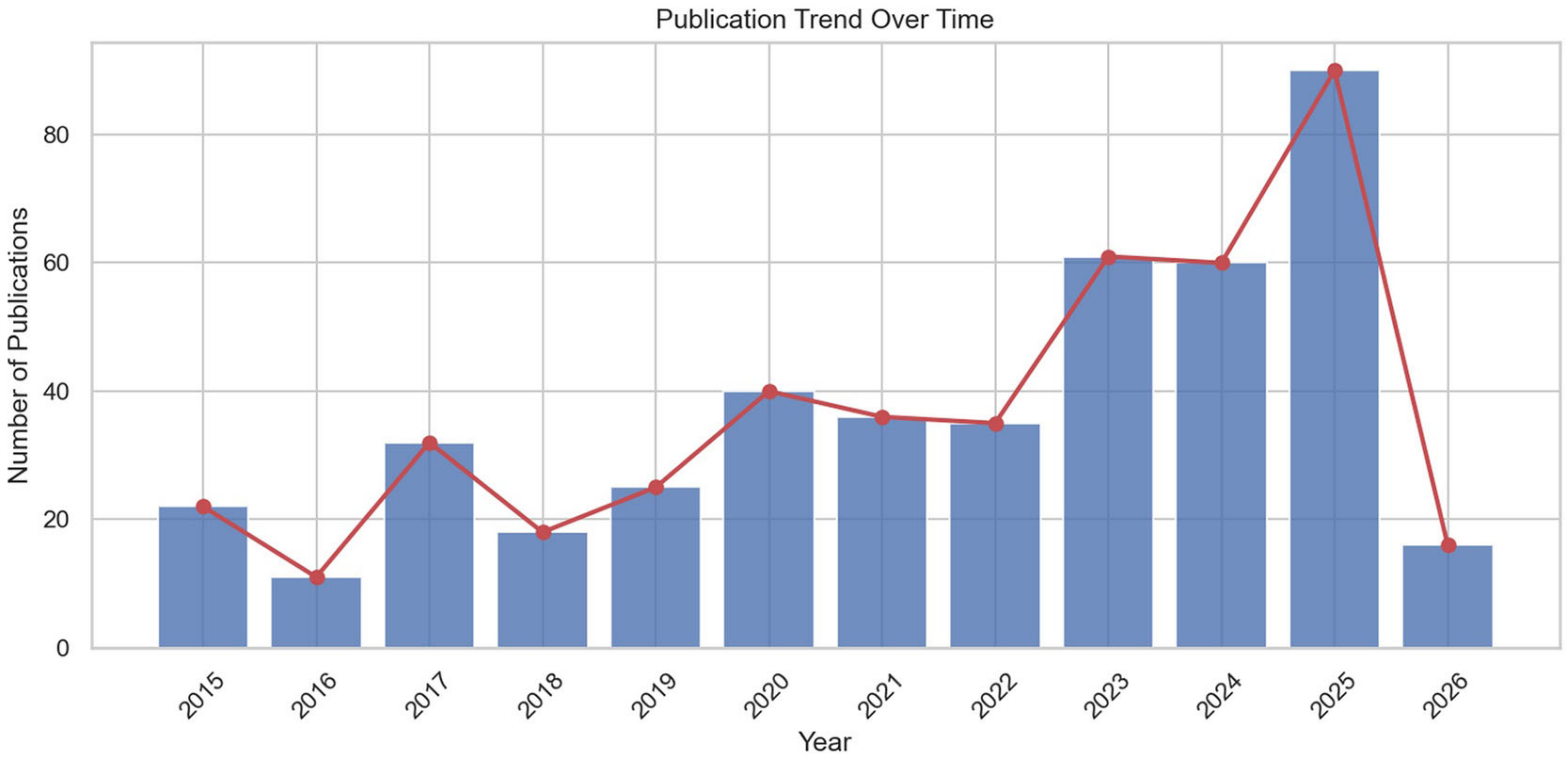
Annual scientific production in the corpus ( *n* = 442, 2015–2026). The temporal distribution shows three distinct phases: initial consolidation (2015–2018), substantial growth coinciding with UNESCO’s ESD for 2030 Roadmap (2019–2022), and exponential acceleration (2023–2026). The single most productive year is 2025 ( *n* = 90).

The systematic search yielded a final corpus of 442 documents spanning the period 2015–2026. The temporal distribution reveals a marked acceleration in scholarly output that can be segmented into three distinct phases (  Figure 4). During the initial phase (2015–2018), production remained modest but grew steadily, with 33 documents in 2015–2016 and 50 in 2017–2018, reflecting an early consolidation of the field following the adoption of the 2030 Agenda. The intermediate phase (2019–2022) witnessed substantial growth, from 65 documents in 2019–2020 to 71 in 2021–2022, coinciding with the publication of UNESCO’s *ESD for 2030 Roadmap* and the global mobilization around SDG 4.7. The acceleration phase (2023–2026) confirms exponential expansion, with 121 documents in 2023–2024 and 106 already registered for 2025–2026—the latter figure being provisional given ongoing indexation. The single most productive year is 2025, with 90 documents, underscoring the field’s continued momentum. Overall, the corpus exhibits a compound annual growth rate that accelerated notably after 2019, with a relative research interest index rising from 0.049 in 2015 to 0.202 in 2025.

The citation landscape is anchored by a small number of foundational publications that have accumulated disproportionate influence. The corpus accrued 10,332 total citations, yielding a mean of 23.17 citations per document and an h-index of 48.  Table 1 presents the ten most-cited documents, which collectively define the field’s empirical and conceptual foundations. Notably, W. L. Filho et al. (2015) leads with 646 citations, followed by Boldureanu et al. (2020) with 590 and Cebrián & Junyent (2015) with 454. The temporal distribution of these landmark works is revealing: the period 2015–2020 contributes all ten entries, with 2015 and 2020 each providing three—a pattern that reflects the convergence of foundational handbooks and SDG implementation frameworks during this period.

**Table 1.** Ten most-cited documents in the corpus.

Rank	Document	Title (abbreviated)	Journal	Citations
1	W. L. Filho et al. (2015)	The future we want: Key issues on SD in HE	*Int. J. Sustain. High. Educ.*	646
2	Boldureanu et al. (2020)	Entrepreneurship education through successful models in HEIs	*Sustainability*	590
3	Cebrián & Junyent (2015)	Competencies in ESD: student teachers’ views	*Sustainability*	454
4	Barth et al. (2015)	Routledge Handbook of HE for SD	*Routledge*	406
5	B. Franco et al. (2019)	Higher education for SD: actioning the global goals	*Sustainability Science*	298
6	W. Filho et al. (2017)	Identifying and overcoming obstacles to SD at universities	*J. Integr. Environ. Sci.*	268
7	Albareda-Tiana et al. (2018)	Implementing the SDGs at university level	*Int. J. Sustain. High. Educ.*	258
8	W. L. Filho et al. (2020)	Sustainability leadership in HEIs: challenges	*Sustainability*	254
9	Ferguson & Roofe (2020)	SDG 4 in higher education: challenges and opportunities	*Int. J. Sustain. High. Educ.*	211
10	Al-Naqbi & Alshannag (2018)	Status of ESD and sustainability knowledge in UAE	*Int. J. Sustain. High. Educ.*	188

*Note.* Citation counts retrieved from Scopus/WoS at the time of analysis.

### Geographic, Institutional, and Disciplinary Distribution

The geographic analysis reveals a predominantly European concentration, with Spain leading the corpus ( *n* = 63 articles, 14.1%), followed by the United Kingdom ( *n* = 52, 11.7%) and Germany ( *n* = 40, 9.0%). The United States ( *n* = 27, 6.1%) and Brazil ( *n* = 17, 3.8%) represent the strongest non-European contributions, while Portugal ( *n* = 16), South Africa and Sweden ( *n* = 14 each), Italy ( *n* = 13), and Colombia ( *n* = 12) complete the top ten. The dominance of Iberian and Germanic countries, combined with meaningful Latin American and African participation, delineates a field whose intellectual center of gravity resides in Western Europe but with increasingly diversified global engagement. Eighty-six countries and 526 institutions are represented across the corpus, confirming the transnational character of ESD research.

**Figure 5.**
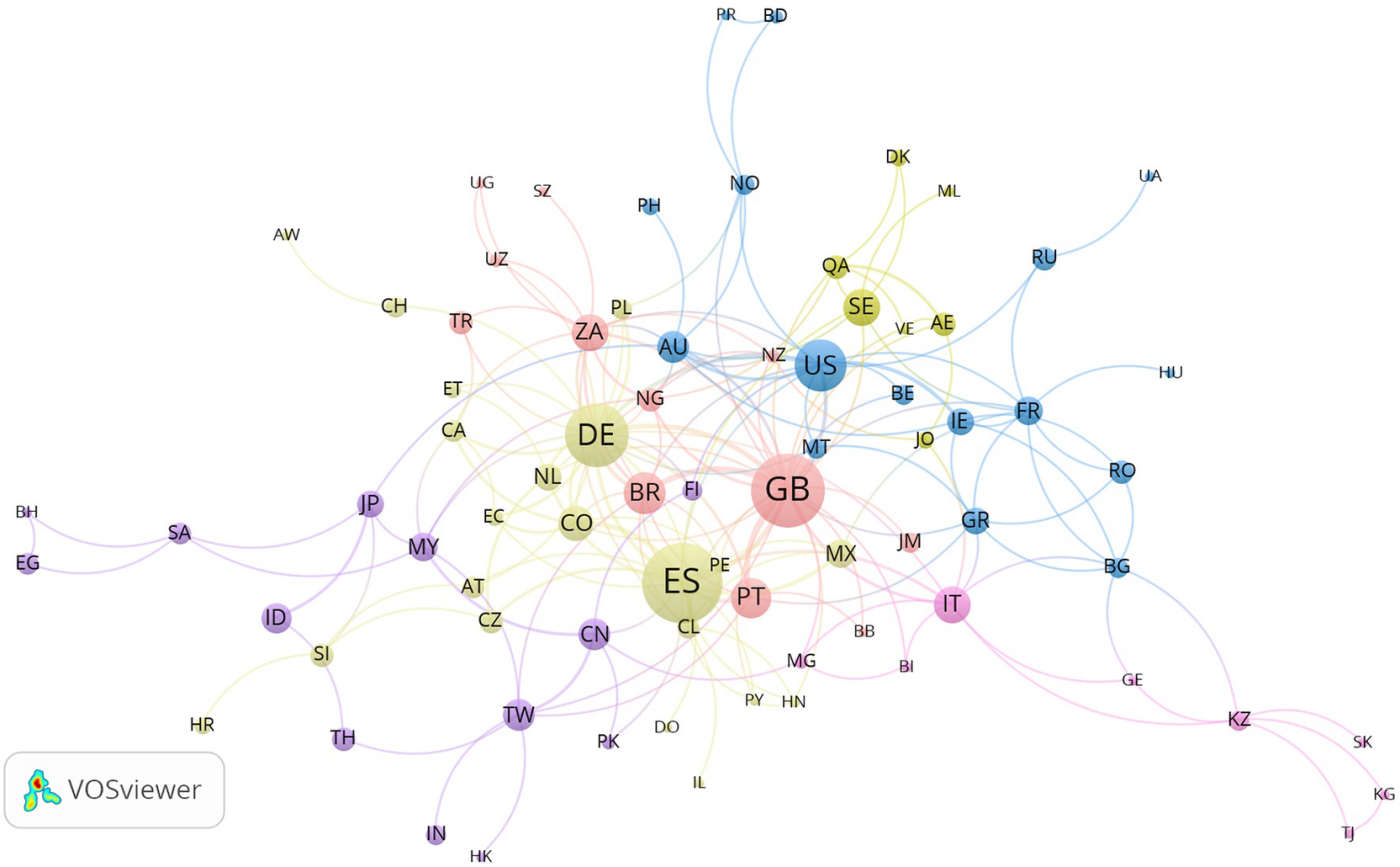
Co-authorship network by countries generated with VOSviewer 1.6.20. Nodes are sized proportionally to the number of documents per country; colors indicate cluster membership; link thickness is proportional to co-authorship strength. The network comprises 86 nations organized into 15 clusters. The United Kingdom occupies the most connected position (30 links, TLS = 55), followed by Germany (22 links, TLS = 39) and Spain (19 links, TLS = 36). Nine countries remain as isolates.

The co-authorship network by countries (  Figure 5) identifies 86 nations organized into 15 clusters. The United Kingdom occupies the most connected position with 30 co-authorship links and a total link strength (TLS) of 55, functioning as the primary hub for international collaboration. Germany follows with 22 links (TLS = 39), maintaining strong ties to Spanish-speaking and Latin American partners. Spain ranks third in collaborative connectivity (19 links, TLS = 36) despite leading in absolute publication volume—a finding that underscores Spain’s strength as a national producer rather than as an international broker. A notable Nordic–Gulf cluster emerges around Sweden, Denmark, Finland, and Qatar, while the Ibero-American cluster connects Spain, Colombia, Mexico, and Portugal through shared linguistic and institutional networks. Nine countries remain isolates with no co-authorship links, revealing persistent gaps in the field’s global integration.

The bibliographic coupling analysis at the institutional level reveals that the most intellectually interconnected institutions are Universitat Hamburg (TLS = 2,812), Universidade Aberta (TLS = 2,375), Manchester Metropolitan University (TLS = 2,345), HAW Hamburg (TLS = 2,053), and Universidade Nova de Lisboa (TLS = 2,032). These institutions, organized into 41 clusters across 502 nodes, form distinct collaborative axes: a German sustainability science axis anchored by Hamburg institutions and Leuphana University of Luneburg (TLS = 1,517); a Portuguese–Brazilian network centered on Universidade Aberta, Universidade Nova de Lisboa, and Universidade Federal de Santa Maria; and a UK education cluster around Manchester Metropolitan University. Universidad de Salamanca (TLS = 1,508) bridges the Spanish-speaking network with the broader European core.

The disciplinary distribution, extracted from 137 unique discipline classifications, confirms the field’s interdisciplinary character. Teacher Education leads ( *n* = 18), followed by Engineering ( *n* = 12), Business ( *n* = 11), and Geography ( *n* = 9), while early childhood education, environmental engineering, and science education contribute smaller but distinctive streams. The ratio of unique disciplines to total articles (137/442 = 0.31) indicates that nearly one-third of all articles self-identify within distinct disciplinary frameworks—a hallmark of a transdisciplinary field still negotiating its boundaries.

### Journal Landscape and Core Publication Venues

The journal co-citation analysis identifies 1,150 sources organized into 105 clusters, revealing a highly differentiated yet hierarchically structured publication landscape. A clear two-journal dominance pattern emerges: the *International Journal of Sustainability in Higher Education* (IJSHE) leads with a TLS of 4,240 and 230 co-citations, followed closely by *Sustainability* (Switzerland) with a TLS of 3,979 and 200 co-citations. Notably, the *Journal of Cleaner Production* ranks third (TLS = 3,960, 210 co-citations), occupying a nearly equivalent position that signals the field’s strong connection to applied sustainability science. *Environmental Education Research* (TLS = 2,837) and *Sustainability Science* (TLS = 2,074) complete the top five. The bibliographic coupling of journals (161 journals, 31 clusters) corroborates this structure, with *Sustainability* (TLS = 1,544) and IJSHE (TLS = 1,464) jointly anchoring the core cluster (  Table 2).

**Table 2.** Top ten journals by co-citation frequency in the network.

Rank	Journal	Co-cit.	TLS	Cluster
1	*Int. J. Sustain. High. Educ.*	230	4,240	1
2	*J. Clean. Prod.*	210	3,960	1
3	*Sustainability (Switzerland)*	200	3,979	20
4	*Environ. Educ. Res.*	150	2,837	4
5	*Sustain. Sci.*	113	2,074	1
6	*J. Educ. Sustain. Dev.*	90	1,412	1
7	*World Sustain. Ser.*	63	922	1
8	*J. Environ. Educ.*	51	827	4
9	*Higher Education*	48	781	11
10	*Sustain. Dev.*	44	737	1

*Note.* TLS = total link strength in the co-citation network.

Three distinct cluster families warrant attention. Cluster 1 forms the core ESD–higher education nexus, anchoring IJSHE alongside *Journal of Cleaner Production*, *Sustainability Science*, and the *World Sustainability Series.* Cluster 4 concentrates environmental and science education journals, including *Environmental Education Research* and *The Journal of Environmental Education*, reflecting the legacy of environmental education research that preceded and now intersects with ESD. Cluster 11, centered on *Higher Education*, represents the higher education policy and governance interface.

### Intellectual Structure: Co-citation and Bibliographic Coupling Analysis

**Figure 6.**
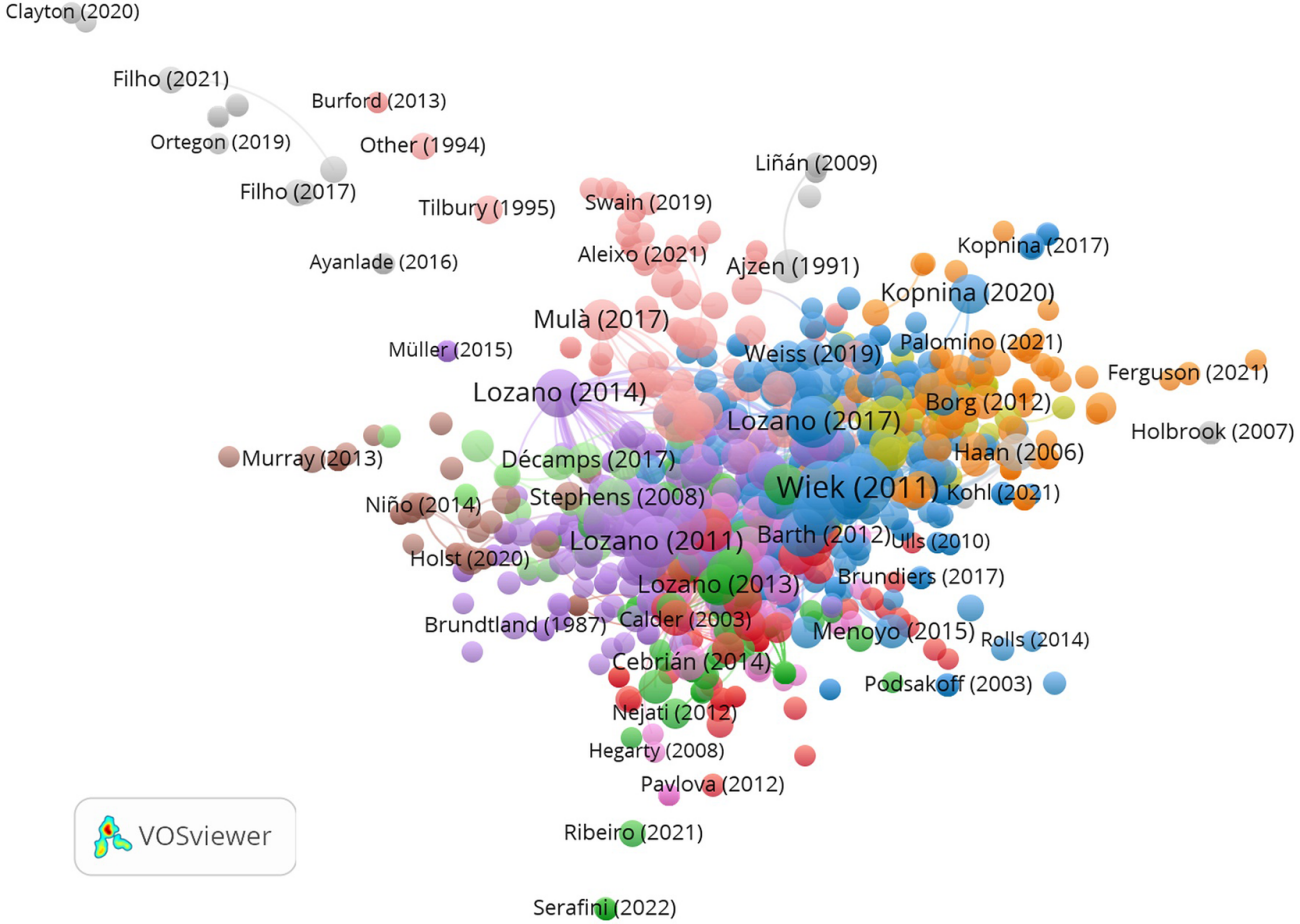
Reference co-citation network generated with VOSviewer 1.6.20. Cluster visualization showing the intellectual pillars of the field. The network encompasses 1,067 cited references organized into 54 clusters. Node size is proportional to co-citation frequency; colors indicate thematic clusters. Wiek et al. (2011) emerges as the most co-cited reference (TLS = 1,082; 66 co-citations).

The reference co-citation analysis identifies 1,067 cited references organized into 54 clusters, revealing the field’s intellectual foundations. Wiek et al. (2011) emerges as the single most co-cited reference (TLS = 1,082; 66 co-citations), establishing key competencies in sustainability as the foundational framework for the entire field. This seminal reference anchors Cluster 3—the largest cluster with 241 nodes—which also encompasses Barth et al. (2007; TLS = 483), Sipos et al. (2008; TLS = 463), and Lozano et al. (2017; TLS = 356), collectively constituting the competence-framework tradition (  Figure 6). Lozano emerges as the most recurrent author in the co-citation network, with four distinct publications appearing among the ten most co-cited references (2006, 2011, 2014, 2017), spanning from institutional transformation to competence operationalization.

The co-citation structure reveals five intellectual pillars:

1. **Competence frameworks and sustainability key competencies** (Cluster 3): Wiek et al. (2011), Barth et al. (2007), Sipos et al. (2008), Lozano et al. (2017), Brundiers et al. (2010, 2020), UNESCO (2017, 2020).
2. **Institutional transformation and whole-institution approaches** (Cluster 2): Wals (2013; TLS = 356), Pauw et al. (2015), O’Flaherty and Liddy (2017).
3. **Curriculum integration and ESD embedding** (Cluster 4): Lambrechts et al. (2012), Mula et al. (2017), Mochizuki and Fadeeva (2010), Bertschy et al. (2013).
4. **Campus sustainability and assessment** (Cluster 5): Lozano (2011; TLS = 422), Lozano (2006; TLS = 300), Lozano (2014; TLS = 314).
5. **Environmental behavior and attitudes** (Cluster 7): Kollmuss and Agyeman (2002), Shephard (2008), Kopnina (2014).

Bibliographic coupling analysis of the 442 documents (369 of which met the minimum citation threshold) further corroborates this structure. The highest-coupled documents— Albareda-Tiana et al. (2018) (TLS = 427), Faham et al. (2017) (TLS = 396), and Brandt et al. (2022; TLS = 351)—share extensive reference lists focused on competence orientation and SDG implementation. Critically, citation count and coupling strength do not always correlate: W. L. Filho et al. (2015), the most-cited document (646 citations), exhibits relatively low coupling strength (TLS = 34), indicating that it is widely cited but draws on a reference base distinct from the field’s mainstream. Conversely, Disterheft et al. (2016) (TLS = 350) and Brassler & Sprenger (2021) (TLS = 348) occupy highly coupled positions despite moderate citation counts, suggesting their role as intellectual bridges within the network.

### Conceptual Structure: Keyword Co-occurrence Analysis

**Figure 7.**
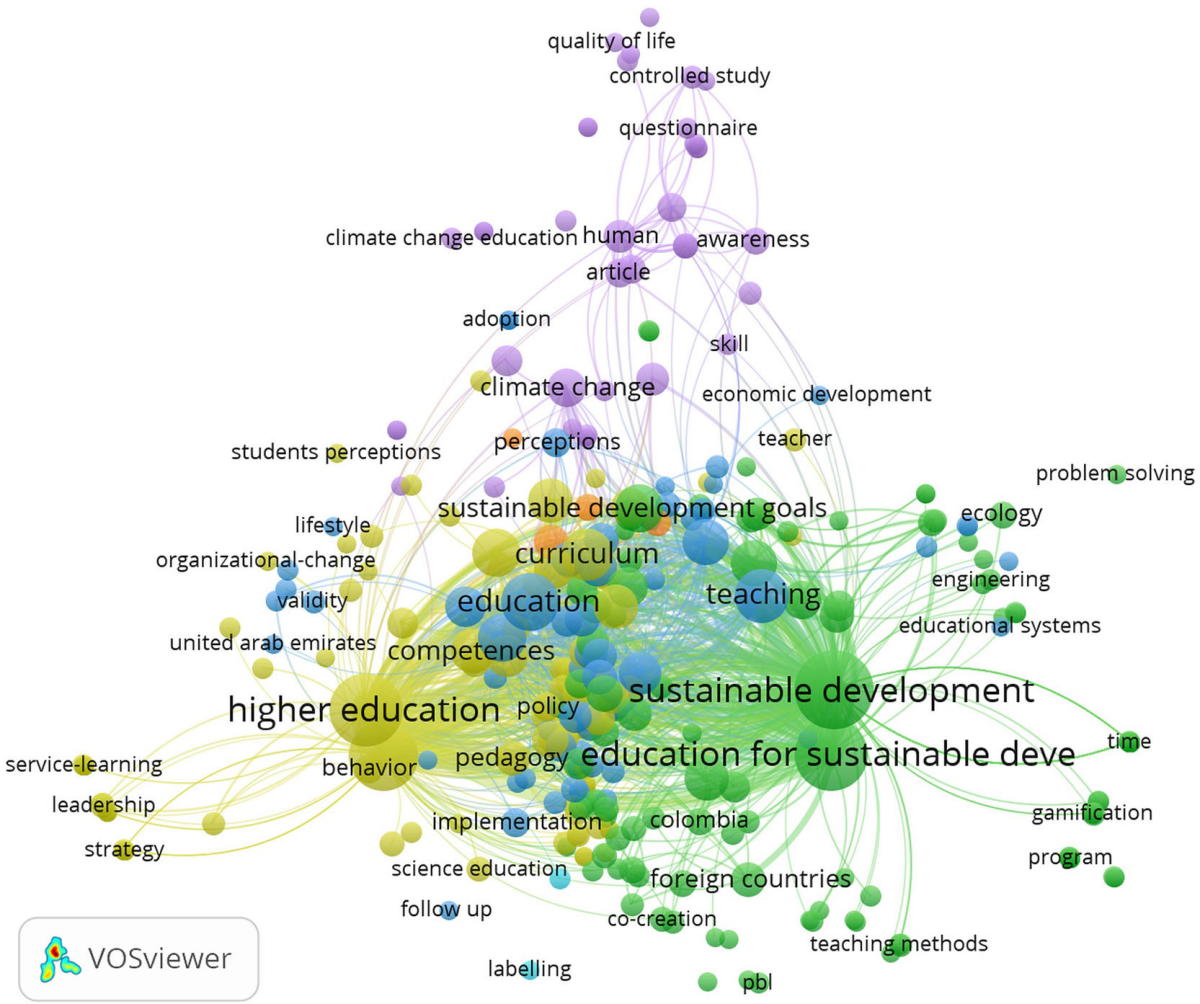
Keyword co-occurrence network generated with VOSviewer 1.6.20. Cluster visualization of 352 author keywords organized into seven thematic clusters. Node size is proportional to keyword frequency; link thickness is proportional to co-occurrence strength. The three most central keywords are *sustainable development* (TLS = 1,204; 166 occurrences), *higher education* (TLS = 833; 133 occurrences), and *sustainability* (TLS = 705; 110 occurrences).

The keyword co-occurrence network (  Figure 7) encompasses 352 keywords organized into seven thematic clusters, offering a fine-grained map of the conceptual territory. The three most central keywords are *sustainable development* (TLS = 1,204; 166 occurrences), *higher education* (TLS = 833; 133 occurrences), and *sustainability* (TLS = 705; 110 occurrences), followed by *education for sustainable development* (TLS = 652; 131 occurrences). The strongest dyadic co-occurrence links— *sustainable development*– *higher education* (weight = 63) and *sustainable development*– *education for sustainable development* (weight = 58)—confirm that the field’s conceptual nucleus remains firmly anchored in the ESD–higher education nexus.

Seven clusters delineate distinct yet interconnected conceptual territories:

**Cluster 1 (Sustainability – Higher Education – Curriculum)** revolves around *sustainability*, *higher education*, and *curriculum*, capturing the normative and structural dimensions of sustainability integration. Keywords such as *key competences*, *systems thinking*, and *experiential learning* indicate the operationalization of competence frameworks into curricular practice.

**Cluster 2 (ESD Implementation and SDG Alignment)** centers on education for sustainable development, sustainable development goals, and sustainable development, incorporating transformative learning, service learning, and design thinking. This is the largest cluster (124 nodes), capturing the implementation-oriented research that connects policy frameworks to pedagogical practice.

**Cluster 3 (Education, Teaching, and Student Behavior)** features education, teaching, learning, knowledge, attitudes, and behavior, constituting the core behavioral research cluster. This cluster captures the empirical tradition of measuring student-level outcomes through surveys and attitudinal instruments ( Álvarez-García et al., 2019; Badea et al., 2020).

**Cluster 4 (Climate Change and Health Education)** is anchored by *climate change* and *climate change education*, with a mean publication year of approximately 2023—the most recent cluster average. The inclusion of nursing-related keywords reveals a discipline-specific pathway for climate literacy that bridges health and environmental education ( Álvarez-Nieto et al., 2018; Bishr et al., 2026).

**Cluster 5 (Engineering, Interdisciplinarity, and Digital Learning)** unites *curricula*, *engineering education*, * e -learning *, *project-based learning*, and *interdisciplinarity*, reflecting the technologically mediated strand of ESD research. The presence of *circular economy* and *massive open online course* keywords reveals emerging conceptual intersections.

**Cluster 7 (Vocational Education and Green Skills)** contains only four nodes but occupies a strategically important bridging position connecting vocational training with the ESD implementation cluster. The keyword *green skills* signals its conceptual proximity to the growing workforce readiness agenda ( Carstensen et al., 2025; Costa et al., 2025).

The density visualization reveals that the highest-density area concentrates around the *sustainable development*– *higher education*– *education for sustainable development* triangle, while lower-density peripheral areas include *climate change education*, *vocational education*, *green skills*, and *circular economy*—terms that, while growing rapidly, remain underrepresented relative to the ESD core.

### Social Structure: Collaboration Patterns

The co-authorship network by authors reveals a characteristic pattern of fragmented collaboration: research teams are predominantly small and largely independent, with no single dominant team spanning the entire field. The institutional co-authorship network (526 nodes, 200 clusters) exhibits extreme fragmentation, with an average cluster size of only 2.6 nodes and 107 institutions (20.3%) remaining as isolates with no co-authorship links. The largest co-authorship cluster centers on Portuguese, Brazilian, and German universities—Universidade Aberta, Universitat Hamburg, HAW Hamburg, and Manchester Metropolitan University—reflecting a Lusophone–Germanic collaborative axis ( Caeiro & Miranda Azeiteiro, 2020; W. L. Filho et al., 2020). A German sustainability education cluster connects Leuphana University with Eberswalde University for Sustainable Development, while a Spanish–Latin American cluster bridges Universidad de Salamanca with Colombian and Mexican institutions.

This fragmented collaboration structure, while indicative of a diverse and distributed research community, also signals limited cross-regional and cross-paradigmatic exchange—a structural feature that may partially explain the conceptual fragmentation between ESD, climate literacy, and green skills traditions identified in the keyword analysis.

### Population, Methodological, and Instrumental Characterization

The population analysis ( *n* = 442 articles) reveals that students constitute the dominant study population. Aggregating all student-related categories—undergraduate students ( *n* = 43, 9.6%), university students ( *n* = 58, 13.0%), higher education students ( *n* = 28, 6.3%), business students ( *n* = 9, 2.0%), and engineering students ( *n* = 8, 1.8%)—yields approximately 146 articles (32.7%) focused on the student experience. Articles classified under the broader category of “Higher Education Context” account for 73 (16.4%), typically describing institutional settings without specifying a discrete study population. Faculty and educator categories collectively account for 31 articles (7.0%), comprising university educators ( *n* = 13), university faculty ( *n* = 12), and teacher educators ( *n* = 6). Pre-service teachers represent a distinctive population ( *n* = 21, 4.7%), bridging the student and educator domains (  Table 3).

**Table 3.** Population categories distribution (top 10).

Population category	*n*	%
Higher Education Context	73	16.4
University Students	58	13.0
Undergraduate Students	43	9.6
Higher Education Students	28	6.3
Pre-service Teachers	21	4.7
University Educators	13	2.9
University Faculty	12	2.7
Business Students	9	2.0
Higher Education Institutions	9	2.0
Engineering Students	8	1.8

Research designs exhibit a similarly concentrated pattern. Survey designs lead ( *n* = 87, 19.5%), followed by case studies ( *n* = 81, 18.2%), qualitative approaches ( *n* = 50, 11.2%), and quantitative studies ( *n* = 43, 9.6%). Mixed-methods designs account for 30 articles (6.7%), while interview-based studies contribute 26 (5.8%). Experimental and quasi-experimental designs together account for only 18 articles (4.0%), reflecting the field’s limited engagement with controlled interventional research—a critical gap for establishing causal evidence on pedagogical effectiveness. Content analysis ( *n* = 23) and systematic reviews ( *n* = 13) represent the documentary and meta-analytic traditions, respectively (  Table 4).

**Table 4.** Research design distribution.

Research design	*n*	%
Survey	87	19.5
Case Study	81	18.2
Qualitative	50	11.2
Quantitative	43	9.6
Mixed Methods	30	6.7
Interview-based	26	5.8
Content Analysis	23	5.2
Action Research	15	3.4
Experimental	14	3.1
Systematic Review	13	2.9
Quasi-experimental	4	0.9

Among reported sample sizes ( *n* = 139 studies, 31.2% of the corpus), the median was 203 and the mean 1,576.2, with a range from 5 to 90,000. The pronounced right skew (mean/median ratio = 7.8:1) indicates that the evidence base is dominated by small-to-medium-scale studies, with a small number of large-scale surveys inflating the mean.

The research focus distribution (  Table 5) reveals that Curriculum Design and Integration ( *n* = 81) leads the scholarly agenda, followed by Institutional Policy and Strategy ( *n* = 53), Teaching Methods and Pedagogy ( *n* = 45), and Framework and Model Development ( *n* = 40). Professional Development ( *n* = 36) and Learning Outcomes and Effectiveness ( *n* = 31) form a second tier. The emergence of Review and Bibliometric Analysis as a distinct focus ( *n* = 31) reflects the field’s growing reflexive awareness. Critically underrepresented areas include Technology and Digital Learning ( *n* = 13) and Interdisciplinary and Systems Thinking ( *n* = 5).

**Table 5.** Research focus distribution.

Research focus	*n*	%
Curriculum Design & Integration	81	18.2
Institutional Policy & Strategy	53	11.9
Teaching Methods & Pedagogy	45	10.1
Framework & Model Development	40	9.0
Professional Development	36	8.1
Learning Outcomes & Effectiveness	31	7.0
Review & Bibliometric Analysis	31	7.0
Competencies & Skills	26	5.8
Assessment & Evaluation	24	5.4
Student Perceptions & Attitudes	22	4.9

The most commonly employed instruments are questionnaires ( *n* = 61) and surveys ( *n* = 38), which together account for 99 articles—confirming the field’s reliance on self-report instruments. Interviews constitute the third most frequent tool ( *n* = 28), followed by content analysis ( *n* = 14) and case studies ( *n* = 12). Project-based learning stands as the most frequently documented pedagogical intervention ( *n* = 11), as exemplified by Fuertes Camacho et al. (2019), Cottafava et al. (2019), and Ariza and Olatunde-Aiyedun (2023).

### Inductive Thematic Synthesis

The inductive thematic analysis of 442 abstracts, conducted through iterative coding across 15 analytic batches using large language model-assisted extraction with systematic hallucination control, yielded five consolidated themes (  Table 6). The overall alignment score was 92%, with 37 hallucination flags identified and subjected to human expert review, indicating robust fidelity between extracted themes and source abstracts.

**Table 6.** Consolidated thematic taxonomy.

Theme	Sub-themes	Robustness
1. ESD Integration in Curricula	(a) Curriculum reform and subject-specific integration; (b) Barriers to ESD implementation	High
2. Transformative Pedagogies and Experiential Learning	(a) Student engagement through experiential learning; (b) Transformative and transdisciplinary pedagogies	High
3. Institutional and Faculty Roles in Advancing ESD	(a) Faculty and institutional leadership; (b) Educator transformation and professional development	High
4. Sustainability Literacy, Values, and Behavioral Outcomes	(a) Sustainability literacy and assessment; (b) Value-based learning and pro-environmental behavior	High
5. Digital Innovation in ESD Delivery	(a) Digital and virtual learning environments; (b) Emerging technologies in sustainability education	High

Theme 1 (ESD Integration) captures studies focused on embedding sustainability principles into curricular structures, including SDG alignment ( Ahmad et al., 2023; Balan et al., 2025) and the persistent challenges of institutional resistance and faculty awareness gaps ( Acosta-Castellanos & Queiruga-Dios, 2022). Theme 2 (Transformative Pedagogies) spans active learning strategies such as project-based learning ( Du et al., 2022), experiential approaches including service learning and community engagement ( Álvarez-Vanegas & Volante, 2024), and technology-mediated instruction encompassing serious games ( Cravero et al., 2021) and augmented reality ( Czok et al., 2023). Theme 3 (Institutional Roles) documents the enabling conditions for curricular transformation, finding that teacher training programs precede and facilitate institutional change ( Biasutti et al., 2018). Theme 4 (Sustainability Literacy) addresses the acquisition of knowledge, attitudes, behaviors, and values as educational outcomes, measured through instruments such as sustainability literacy tests and pre–post evaluations. Theme 5 (Digital Innovation), the most temporally recent, reflects the growing role of virtual reality, digital twins, artificial intelligence, and online academies in ESD delivery.

The cross-analysis identified three recurring inter-thematic configurations: (1) an **Integrated Education Design** configuration linking curriculum, pedagogy, and competencies as a coherent teaching–learning system; (2) an **Institutional Support System** linking organizational strategies with educator development as enabling conditions; and (3) a **Technology-Enhanced Learning** trajectory connecting digital innovation with both pedagogical approaches and student outcomes.

### Research Frontiers and Knowledge Gaps

**Figure 8.**
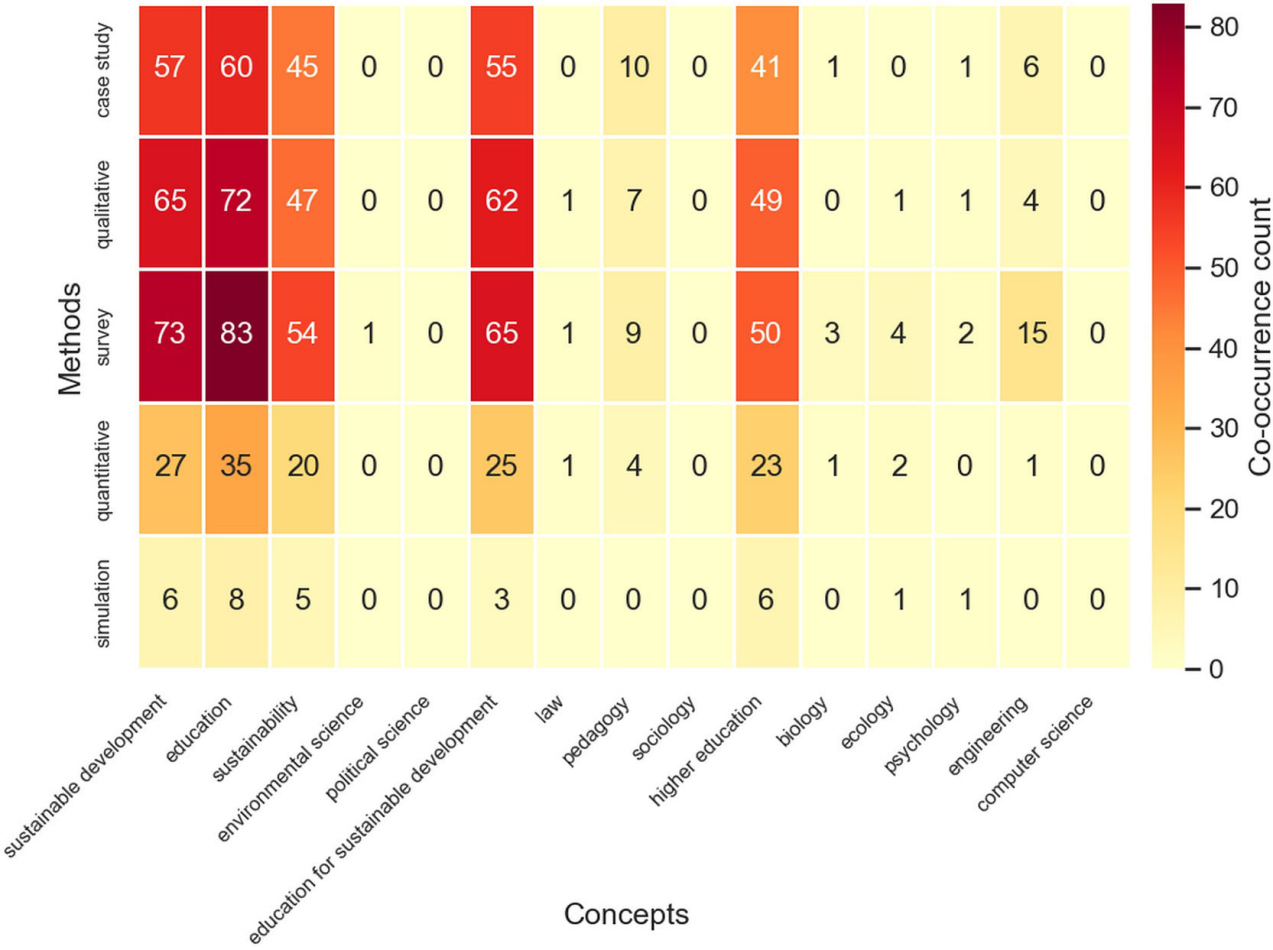
Methods-by-concepts coverage matrix showing 64.0% overall coverage. Each cell represents a unique combination of research design and conceptual domain. Filled cells indicate existing evidence; empty cells indicate structural knowledge gaps where methodologically plausible research combinations remain unexplored. Fifty-six knowledge gaps were identified across structural, declared, and emergent categories.

The state-of-the-art analysis identified 56 knowledge gaps classified into three taxonomic categories: structural gaps ( *n* = 25), declared gaps ( *n* = 23), and emergent gaps ( *n* = 8) (  Figure 8). The coverage analysis of Methods × Concepts matrices revealed 64.0% coverage (48 of 75 cells populated), while Methods × Applications achieved 74.1% coverage, indicating that approximately one-third of methodologically plausible combinations remain unexplored.

**Structural gaps** represent combinations of frequent approaches and domains that never co-occur in the corpus. The qualitative research tradition accounts for multiple structural gaps, being systematically absent from biology, clinical, and medical domains—suggesting that human-dimension research has not penetrated health and natural-science sustainability subfields. The simulation–pedagogy and case study–ecology gaps indicate that innovative methodological approaches have not been systematically paired with emerging content domains.

**Declared gaps**, explicitly articulated by researchers within their abstracts, reveal a convergent pattern of concerns (  Table 7). Multiple studies note the scarcity of longitudinal research on ESD intervention effects ( Brassler, 2025; Collado et al., 2022), the limited investigation of comparative studies between training formats ( Brassler & Schultze, 2021), and the absence of causal models of sustainability competency acquisition ( Abdullahi et al., 2024). Researchers also identify insufficient attention to ESG integration in general education ( Chien & Chien, 2025) and the need for mixed-method approaches to capture institutional change dynamics ( Costa et al., 2025).

**Table 7.** Selected declared knowledge gaps with representative references.

Gap area	Key references
Longitudinal impact assessment of ESD interventions	Brassler (2025); Collado et al. (2022)
Comparison between ESD delivery formats	Brassler & Schultze (2021)
Causal models of sustainability competency acquisition	Abdullahi et al. (2024)
ESG and financial sustainability in curricula	Chien & Chien (2025); Garcia-Hernandez et al. (2025)
Scalability across geographic and institutional contexts	Álvarez-Vanegas & Volante (2024)
Mixed-method approaches for institutional change	Costa et al. (2025)

**Emergent gaps** arise from rapidly growing areas that lack adequate methodological coverage. Simulation-based approaches show the highest growth rate (new entrant with 8 uncovered combinations), yet remain disconnected from pedagogical theory. Quantitative methods (2.36x growth) have seven uncovered combinations with key domains, while medical (2.81x growth) and clinical applications (2.11x growth) present expanding frontiers with insufficient qualitative coverage. The fastest-growing research frontiers include simulation (new), economic growth (3.37x), medical applications (2.81x), pedagogy as a concept (2.39x), and quantitative methods (2.36x).

The convergence of these findings points to a field in active conceptual transition. The traditional ESD paradigm—rooted in competence frameworks, institutional transformation, and curriculum integration—is progressively intersecting with climate-specific constructs (climate literacy, climate action competence), green economy demands (green skills, sustainable entrepreneurship; Garcia-Hernandez et al. (2025); Carstensen et al. (2025)), and digitally mediated pedagogies (simulations, serious games, AI-enhanced learning). Yet the evidence base remains characterized by survey and case-study dominance, limited experimental rigor, geographic Euro-centrism, and fragmented collaboration networks. The structural absence of qualitative research from health and natural-science domains and the emergent but methodologically underdeveloped frontiers of simulation and digital learning constitute the most pressing gaps for the next generation of ESD research in higher education and vocational education contexts.

## Discussion

The bibliometric mapping and inductive thematic analysis of 442 documents spanning 2015–2026 suggest that Education for Sustainable Development (ESD) in higher education has become an established research field with identifiable intellectual pillars, persistent geographic asymmetries, and notable methodological limitations. Five emergent themes—curriculum integration, transformative pedagogies, institutional and faculty roles, sustainability literacy and behavioral outcomes, and digital innovation—collectively delineate a field whose conceptual nucleus remains anchored in the competence-framework tradition ( Wiek et al., 2015) yet is progressively intersecting with climate-specific constructs and green economy demands. This discussion considers the main tensions, structural patterns, and emerging areas that the findings highlight, drawing on recent multidisciplinary evidence.

### Aligning Educational Outcomes with the Green Economy: Geographic Asymmetries and Curricular Gaps

The keyword co-occurrence analysis identified *green skills* and *circular economy* as rapidly growing yet peripheral concepts, occupying low-density areas in the network despite their increasing policy urgency. This peripherality—consistent with the differential marginal contributions observed during search strategy validation, where terms such as *climate literacy* and green skill* proved necessary for capturing otherwise missed literature—suggests a gap between the field’s conceptual core and the workforce demands of the green transition. As nations pursue decarbonization and circular economy models, higher education institutions (HEIs) face growing expectations to prepare graduates with competencies in green finance, sustainable industrialization, and climate adaptation ( Desalegn & Tangl, 2022; Tamasiga et al., 2025). Yet our results reveal that this pressure is unevenly distributed: Spain, the United Kingdom, and Germany collectively account for over one-third of all publications, while the Global South—with the notable exceptions of Brazil, South Africa, and Colombia—remains structurally underrepresented. This geographic concentration, corroborated by the co-authorship network where nine countries remain isolates with no collaborative links, points to a gap in current ESD research coverage. Developing nations face distinct challenges that may require localized, context-sensitive educational frameworks rather than imported models ( Dias Paião Júnior & Ferraz, 2024; Ndoka & Leskaj, 2025). Addressing this gap would involve linking ESD more closely to labor market realities, connecting academic sustainability with green skills relevant to regional industrial contexts ( Garcia-Hernandez et al., 2025; St Flour & Bokhoree, 2021).

### Pedagogical Transformation, Sustainability Literacy, and the Measurement Challenge

The dominance of Theme 2 (Transformative Pedagogies) and Theme 4 (Sustainability Literacy and Behavioral Outcomes) in our thematic synthesis confirms that the field has moved decisively beyond transmissive instruction toward experiential, student-centered paradigms. However, a critical tension emerges from the methodological characterization: surveys and case studies together account for nearly 38% of all research designs, while experimental and quasi-experimental designs represent only 4.0% of the corpus. This methodological imbalance limits the field’s capacity to establish causal evidence for pedagogical effectiveness. Several authors argue that complex sustainability problems are difficult to address within monodisciplinary silos, suggesting that HEIs should foster environments where students and external partners co-construct knowledge through real-world engagement ( Kurris et al., 2026; Leal Filho et al., 2025). SDG-aligned projects in STEM fields suggest that experiential learning can enhance interdisciplinary competencies, intercultural awareness, and intrinsic motivation ( Hu, 2026; Johnson, 2026; Poya, 2026; Roy et al., 2025). Embedding climate change adaptation into civil engineering curricula equips future professionals with both technical viability and ethical capacity for resilient infrastructure design ( Baah et al., 2025; Dupuis et al., 2025; Kordi et al., 2025).

Yet the measurement of sustainability literacy—particularly the transition from “knowing” to “doing”—remains a declared knowledge gap across the corpus. Our gap analysis identified the scarcity of longitudinal impact assessments and causal models of competency acquisition as the most recurrently articulated research needs. The concept of “Work 5.0,” combining human-centric principles with advanced technologies, calls for competence frameworks that include critical reflection, digital literacy, and systems thinking ( Zare et al., 2026). This suggests that ESD may need to move beyond knowledge acquisition toward fostering the dispositions and skills that enable graduates to integrate sustainability considerations into their professional practice ( Ferreira De Mello et al., 2026; López et al., 2025).

### The Institutional Architecture: From Declarations to Structural Embeddedness

While pedagogical innovation operates at the micro-level, the institutional environment functions as either catalyst or barrier to ESD implementation. Our results identified extreme fragmentation in the institutional co-authorship network—200 clusters across 526 institutions with 20.3% remaining as isolates—revealing that ESD research is conducted in largely disconnected pockets with limited cross-institutional exchange. This structural fragmentation mirrors qualitative findings from diverse contexts: despite growing international pressure, SDG integration is frequently hampered by weak institutional commitment, resource constraints, and absent implementation structures ( Dalelo, 2026). This suggests that HEIs may need to move beyond declarative sustainability commitments toward embedding sustainability more systematically into governance, campus operations, and curriculum development ( Arnado, 2023; Campos Junges et al., 2026).

An important component of this institutional dimension is faculty development. Our population analysis found that educators and faculty constitute only 7.0% of all study populations, compared to 32.7% for students—a ratio that suggests the field has focused predominantly on learner outcomes, with less attention to the educators responsible for pedagogical delivery. Studies on faculty development interventions, whether delivered through digital platforms or face-to-face modalities, report improvements in self-efficacy, knowledge, and student engagement ( Hemmer et al., 2024; Ravdansuren et al., 2025). From an ecological perspective, educator development is shaped by the interplay of individual capabilities, institutional support, collaboration with external stakeholders, and policy environments ( Bebenimibo & Mutua, 2025; J. Wang & Jiang, 2025). Competency-oriented assessment frameworks—such as the Rounder Sense of Purpose—may help ensure that sustainability education translates into measurable outcomes, particularly in foundational domains like pre-service teacher training ( Balis & Vungthong, 2026; Valderrama-Hernández et al., 2025). Institutional support also directly mediates the relationship between students’ sustainability competencies and their pro-environmental behavioral intentions ( Arnejo et al., 2025), reinforcing the inseparability of institutional infrastructure and individual learning outcomes.

### Digital Innovation and the Emergent Research Frontier

Our keyword analysis positioned *e-learning *, *massive open online courses*, and *serious games* within the engineering and interdisciplinarity cluster, while the state-of-the-art gap analysis identified simulation-based approaches as the fastest-growing methodological frontier with the highest number of uncovered domain combinations. This convergence points to digital innovation as a rapidly growing yet methodologically underdeveloped area in ESD research. The emerging “University 5.0” model proposes linking technological adoption to inclusivity, equity, and environmental responsibility ( Morales-Arevalo et al., 2025). Digital workflows and virtual collaborative environments offer opportunities for expanding access and reducing carbon footprints ( Eisinger Balassa & Buics, 2026; Gavrus et al., 2025), while innovative applications such as telepresence robots with gamification elements boost engagement among geographically isolated demographics ( Addas et al., 2024). Yet the literature sharply cautions against uncritical deployment: digitalization can exacerbate divides, generate electronic waste, and produce hidden environmental costs through energy-intensive data processing. In conflict-affected or resource-constrained regions, however, digital transformation acts as a vital mechanism for academic resilience ( Al-Shaer et al., 2025).

Our coverage matrix revealed that the qualitative research tradition is systematically absent from clinical, medical, and biological sustainability domains—a structural gap that digital and simulation-based methods are uniquely positioned to address. The integration of virtual reality, digital twins, and AI-enhanced learning environments into ESD could offer new possibilities for investigating competency acquisition in domains where field experimentation is ethically or logistically constrained. This suggests that HEIs would benefit from approaching digital transformation with sustainability considerations in mind ( Trevisan et al., 2024).

Taken together, these findings suggest that further development of ESD in higher education involves multiple interconnected dimensions: aligning curricula with green economy needs, strengthening institutional support mechanisms, using digital transformation responsibly, and investing in faculty development. The 56 knowledge gaps identified in this review offer a structured starting point for future research planning.

## Conclusions

This study mapped the intellectual, social, and conceptual structure of Education for Sustainable Development in higher and vocational education by integrating bibliometric network analysis with artificial intelligence-assisted inductive thematic synthesis. The analysis of 442 documents spanning 2015–2026 yielded several findings that complement prior reviews, which have typically focused on specific regions, sub-disciplines, or implementation practices.

First, the temporal and geographic profiling established that the field has undergone exponential growth since 2019, yet this expansion is concentrated in Western Europe—with Spain, the United Kingdom, and Germany accounting for over one-third of all output—while the Global South remains structurally underrepresented despite its disproportionate exposure to sustainability challenges. The co-authorship network further revealed that nine countries and over 20% of institutions operate in complete isolation, signaling that the field’s growth is extensive rather than integrative.

Second, the intellectual structure analysis uncovered a field organized around five co-citation pillars anchored by the competence-framework tradition, with Wiek et al. (2011) as the foundational reference. The journal landscape is governed by a clear two-journal core—the *International Journal of Sustainability in Higher Education* and *Sustainability* —yet the emergence of *Journal of Cleaner Production* as a third gravitational center reveals the field’s progressive connection to applied sustainability science. The keyword co-occurrence network of 352 terms organized into seven clusters confirmed that while the conceptual nucleus remains anchored in the ESD–higher education nexus, peripheral areas including climate literacy, green skills, and vocational education are expanding rapidly without yet achieving structural integration with the core.

Third, the methodological characterization exposed a critical evidence gap: surveys and case studies dominate the research landscape, while experimental and quasi-experimental designs account for only 4.0% of all studies. The population focus is overwhelmingly student-centered, with faculty and educators representing a mere 7.0% of study populations—an asymmetry that constrains understanding of the very agents responsible for curricular transformation. Sample sizes are predominantly small to medium, and nearly one in five articles fails to report its study population, reflecting a persistent reporting deficit.

Fourth, the inductive thematic synthesis—verified at a 92% alignment score with 37 hallucination flags subjected to human expert review—consolidated the discourse into five themes: ESD integration in curricula, transformative pedagogies and experiential learning, institutional and faculty roles, sustainability literacy and behavioral outcomes, and digital innovation in ESD delivery. The cross-thematic analysis revealed three recurring configurations: an integrated education design system linking curriculum, pedagogy, and competencies; an institutional support system connecting organizational strategies with educator development; and a technology-enhanced learning trajectory linking digital tools with pedagogical approaches and student outcomes.

Fifth, the systematic knowledge gap analysis identified 56 gaps classified as structural, declared, and emergent. The coverage matrix of methods by concepts achieved only 64.0% coverage, confirming that approximately one-third of methodologically plausible combinations remain unexplored. The structural absence of qualitative research from health and natural-science sustainability domains, combined with the emergent but methodologically underdeveloped frontiers of simulation and clinical applications, represents a notable gap for future scholarship to address.

### Limitations

Several inherent limitations must be acknowledged. The reliance on Scopus, Web of Science, and ERIC inherently favors English-language, indexed publications and may underrepresent sustainability discourse emerging from regional or non-indexed journals. The exclusion of gray literature, institutional reports, and policy documents limits the capture of practitioner-oriented knowledge. While the LLM-assisted thematic analysis increased the capacity for qualitative synthesis at scale, its interpretive outputs are constrained by the algorithmic architecture of the applied generative models, despite the 92% alignment verification, 37 hallucination flags reviewed, and human expert validation protocols.

### Future Research Directions

The convergence of the identified knowledge gaps points toward four priority axes for future investigation. First, the field urgently requires a methodological pivot toward longitudinal and experimental designs capable of establishing causal evidence for the impact of specific pedagogical interventions on sustained behavioral outcomes. Second, research must amplify scholarship from the Global South, developing context-sensitive frameworks that address the distinct socio-economic barriers to ESD implementation in developing contexts. Third, simulation-based and digitally mediated approaches—the fastest-growing yet most methodologically disconnected frontier—demand systematic pedagogical theorization to move beyond technological novelty toward evidence-based integration. Fourth, the extreme fragmentation of collaboration networks calls for deliberate cross-institutional and cross-regional research consortia capable of generating the comparative, multi-site evidence that the field currently lacks. Addressing these axes could contribute to moving the field from conceptual proliferation toward a more evidence-based approach to sustainability education in the context of the 2030 Agenda.

## Author contributions

Author contributions are reported using the CRediT (Contributor Roles Taxonomy) framework:

• **Segundo Francisco Segura-Altamirano:** Conceptualization, Data Curation, Formal Analysis, Methodology, Software, Visualization, Writing – Original Draft Preparation.
• **Julio Santos Hilario-Vargas:** Data Curation, Formal Analysis, Methodology, Supervision, Validation, Writing – Original Draft Preparation, Writing – Review & Editing.
• **Lindon Vela-Melendez:** Conceptualization, Data Curation, Formal Analysis, Methodology, Software, Validation, Writing – Original Draft Preparation.
• **Carmen Graciela Arbulu-Perez-Vargas:** Investigation, Formal Analysis, Methodology, Supervision, Writing – Original Draft Preparation.
• **Jose Teodoro Reupo-Periche:** Investigation, Resources, Writing – Original Draft Preparation.
• **Jhon Wiston Garcia-Lopez:** Investigation, Resources, Writing – Original Draft Preparation.
• **Mauro Adriel Rios-Villacorta:** Investigation, Formal Analysis, Methodology, Supervision, Writing – Original Draft Preparation.
• **Hugo Javier Chiclayo-Padilla:** Data Curation, Project Administration, Validation, Writing – Review & Editing.
• **Diana Mercedes Castro-Cardenas:** Conceptualization, Data Curation, Formal Analysis, Software, Validation.

## Data Availability

All data underlying this study—including bibliographic datasets ( *n* = 442 articles), enriched metadata, extraction templates, analysis scripts, VOSviewer maps, qualitative codebooks, and thematic synthesis outputs—are openly available in a version-controlled repository. Segura-Altamirano, S. F., Hilario-Vargas, J. S., Vela-Melendez, L., Arbulu-Perez-Vargas, C. G., Reupo-Periche, J. T., Garcia-Lopez, J. W., Rios-Villacorta, M. A., Chiclayo-Padilla, H. J., & Castro-Cardenas, D. M. (2026). *Mapping the Intellectual Architecture of Education for Sustainable Development in Higher Education: A Bibliometric and AI-Assisted Thematic Synthesis—Data and Analysis Repository* (Version 1.0) [Data set]. Zenodo. https://doi.org/10.5281/zenodo.19416905

## Software availability

All computational analyses were conducted in **Python 3.9.7** (Python Software Foundation, https://www.python.org). The key libraries and their versions used were: numpy 2.0.2 and scipy 1.13.1 for numerical computation; matplotlib 3.9.4 and seaborn 0.13.2 for static visualizations; plotly 6.5.2 with kaleido 1.2.0 for interactive and exportable figures; networkx 3.2.1 with python-louvain 0.16 for network construction and community detection; scikit-learn 1.6.1 for clustering and dimensionality reduction; nltk 3.9.2 and wordcloud 1.9.6 for text processing; bibtexparser 1.4.4 for bibliographic record parsing; RapidFuzz 3.13.0 for fuzzy string deduplication; and openpyxl 3.1.5 for spreadsheet handling. The AI-assisted thematic analysis was conducted via the OpenAI-compatible API client (openai 2.24.0) interfacing with Qwen3-235B and DeepSeek V3.2 models. The multi-rater LLM screening platform was developed in-house using Python with tkinter for the graphical interface and SQLite for traceability logging. Bibliometric network visualizations (co-citation, co-authorship, keyword co-occurrence) were generated using **VOSviewer 1.6.20** (Centre for Science and Technology Studies, Leiden University; https://www.vosviewer.com).

• Software available from: https://www.python.org; https://www.vosviewer.com
• Source code available from: https://github.com/sefran1020/Mapp_ESD_HE_Bibliometric
• Archived source code at time of publication: https://doi.org/10.5281/zenodo.19416905
• License: MIT (source code); CC BY 4.0 (data and analysis outputs)
