# Chinese Medicine as First-Line Treatment for Subthreshold Mental Disorders in Primary Care: Opportunities and Challenges

**DOI:** 10.12688/f1000research.163621.2

**Published:** 2025-10-09

**Authors:** Hiu To Tang, Jingyuan Luo, Hoi Ki Wong, Albert Yeung, Danny J. Yu, Zhaoxiang Bian

**Affiliations:** 1 Vincent V.C. Woo Chinese Medicine Clinical Research Institute, School of Chinese Medicine, Hong Kong Baptist University, Hong Kong SAR, China; 2 School of Chinese Medicine, Hong Kong Baptist University, Hong Kong SAR, China; 3 Centre for Chinese Herbal Medicine Drug Development Limited, School of Chinese Medicine, Hong Kong Baptist University, Hong Kong SAR, China; 4 Depression Clinical and Research Program, Massachusetts General Hospital, Boston, USA; 5 The Chinese Medicine Hospital of Hong Kong, Hong Kong SAR, China

**Keywords:** Chinese medicine, Subthreshold mental disorders, Primary care

## Abstract

Subthreshold mental disorders (SMDs), characterized by clusters of psychiatric symptoms that do not meet the criteria for a formal diagnosis yet are sufficiently severe to impair daily functioning. SMDs exhibit a high prevalence and an elevated risk of progression to diagnosed disorders and impose a substantial socioeconomic burden. Despite their significant impact, SMDs often go overlooked and untreated due to a global shortage of mental health professionals and stigmatization associated with conventional psychological and psychiatric treatments. This perspective advocates the integration of Chinese medicine (CM) as a first-line treatment for SMDs, focusing specifically on primary care settings in regions with established CM infrastructure and high public acceptance. Emerging evidence has shown that CM treatments, including acupuncture, herbal medicine, and other modalities, can be effective in managing various mental disorders. Systematic reviews have shown that herbal medicine not only has fewer side effects compared to psychotropic medications but also reduces adverse effects when used as adjunctive therapy. The potential benefits of using CM include mitigating the shortage of mental health professionals by supplementing primary care, preventing the exacerbation of SMDs, and offering a less stigmatized, cost-effective option that could improve help-seeking behaviors. However, challenges such as lack of recognition, insufficient collaboration between CM and mental health specialists, and differing theoretical frameworks hinder its integration into primary care in the mental health care field. Addressing these challenges will require public education, robust research evidence, policy changes, and the development of collaborative frameworks. This study highlights the need for greater recognition and integration of CM as a viable first-line treatment for the management of SMDs within primary care settings.

**Abbreviations**
APA: American Psychological Association
CBT: Cognitive Behavioral Therapy
CHM: Chinese herbal medicine
CI: Confidence interval
CM: Chinese Medicine
DSM: Diagnostic and Statistical Manual of Mental Disorders
HDRS: Hamilton Depression Rating Scale
NICE: National Institute for Health and Care Excellence
OR: Odds ratio
RAR: Rate ratio
RCT: Randomized controlled trial
SMD: Standardized mean difference
SMDs: Subthreshold mental disorders

## 1. Introduction

Subthreshold mental disorders (SMDs) are characterized by clusters of psychiatric symptoms that fall short of meeting full diagnostic criteria for formal mental disorders in terms of symptom count, duration, or severity, yet still lead to significant distress and functional impairment. ^1, 2^ Despite growing recognition of SMDs, the lack of consensus has resulted in terminological diversity within the literature, ^3, 4^ including terms such as “subsyndromal disorders”, ^2^ “subthreshold psychiatric symptoms”, ^5^ and “minor psychiatric disorders”. ^6^ However, this heterogeneity of terminology converges on an intermediary nosological category between normative mental functioning and diagnosable psychiatric disorders. ^7^ These conditions are clinically classified under the “other specified” categories of the Diagnostic and Statistical Manual of Mental Disorders (DSM-5) or the “not otherwise specified” categories of DSM-IV, operationalizing terminological diversity into standardized diagnostic frameworks. ^8– 10^ Numerous epidemiological studies worldwide have consistently demonstrated that SMDs are more common than diagnosed mental disorders in general population and primary care. ^11^ In a meta-analysis, the pooled prevalence of subthreshold depression is reported at 11.1%, ^12^ which exceeds the prevalence of depressive disorders, reported as 6.38% in the general population. ^13^ Similarly, 10% of the Chinese population exhibits symptoms of a subthreshold anxiety disorder, significantly higher than the 3.5% who fulfill the criteria for a clinically diagnosed anxiety disorder. ^14^ SMDs, while not meeting full diagnostic criteria, can still affect both individual functionality and broader socioeconomic development. In a representative sample from the UK, 12.6% of individuals exhibiting SMDs at baseline were found to develop a new functional disability, a rate significantly higher than the 7.7% observed among healthy individuals. ^15^ Additionally, SMDs were responsible for over 32 million days of work lost in the year prior to the study. ^15^ A study in Norway found that about 20% of medical leave episodes and a third of all disability pensions are attributed to SMDs. ^16^ The exacerbation of risk associated with SMDs is particularly evident in the progression to full-blown mental disorders. Specifically, the incidence of major depression was observed at a rate of 17.6% among individuals with subthreshold depression, markedly higher than the 6.1% observed among healthy individuals. ^12^ The high prevalences of SMDs and their impacts on functioning impose a noteworthy socioeconomic burden that should not be disregarded.

Significant challenges exist in managing SMDs in the mental health field. While psychotherapeutic interventions demonstrate a moderate effect size of 0.42 in alleviating SMDs, ^17^ and are recommended as one of the first-line treatment in clinical guidelines, ^18^ the shortage of mental health professionals, especially in primary care where SMDs are most commonly seen, can lead to delayed and underprovided treatment. ^19, 20^ The WHO’s Mental Health Atlas 2020 underscores this issue, reporting a mean of fewer than 3 mental health workers per 100,000 population in the Southeast Asia and African regions. This figure is considerably lower than the global median of 13. ^21^ This scarcity of mental health professionals also manifests in highly developed areas like Hong Kong, where the waiting period for psychiatric appointments in public hospitals can extend beyond two years for mild cases such as SMDs. ^22^ Additionally, the current healthcare paradigm frequently overlooks preventive screening for such cases, resulting in missed opportunities for early intervention. ^23^ Active monitoring is recommended for SMDs in the National Institute for Health and Care Excellence (NICE) clinical guidelines. ^24, 25^ However, studies have shown that active monitoring may be insufficient compared to proactive treatment in subthreshold states. ^26^ Initiating pharmacological interventions at these early and subthreshold stages may be considered premature and can lead to treatment failures with no significant difference in outcomes compared to placebo. ^27^ Another challenge is the often delayed or completely absent help-seeking, which exacerbates the problem. ^28, 29^ Stigma is one of the most significant contributors to this issue. A meta-analysis has shown that stigma related to mental health services is directly associated with less active help-seeking for mental problems in the general population (Odds ratio (OR) = 0.80, 95% Confidence interval (CI) 0.73–0.88). ^30^ Cultural familiarity also plays a significant role in healthcare decisions. Although Cognitive Behavioral Therapy (CBT) is effective, it was originally developed in a Western context and exhibits smaller effect sizes in Chinese populations, ^31^ and its effectiveness is reduced without cultural adaptation. ^32^ Therefore, it is imperative to identify alternative treatments for SMDs that are effective, offer sufficient workforce provision and are less stigmatized compared to conventional treatments. In confronting these challenges, Chinese medicine (CM) presents as one of the promising first-line treatment options in primary care for the management of SMDs, particularly within primary care systems that have existing infrastructure and high public acceptance, such as those in mainland China and Hong Kong.

## 2. Chinese Medicine and Subthreshold Mental Disorders

CM, with its roots spanning thousands of years, has a rich history of treating mental disorders. ^33^ It has been incorporated into the healthcare system in numerous Asian countries and viewed as a complementary medical system in many Western nations. ^34, 35^ CM aims to rectify imbalances and restore patients’ holistic wellness, encompassing both the physical and mental aspects of patients’ health. ^36^ Several CM practices are recognized for their potential in preventing and treating mental disorders. For instance, acupuncture is considered as adjunctive treatment to antidepressant medication for depression by the American Psychological Association (APA). ^37^ Chinese herbal medicine and acupuncture are recommended to treat insomnia in the Hong Kong Chinese Medicine Clinical Practice Guideline. ^38^ The NICE has also reviewed the efficacy of combining acupuncture with antidepressants for depression. ^24^ Moreover, various CM treatments, including herbal medicine, acupuncture, cupping, and tuina, are endorsed for treating anxiety in clinical guidelines published by the National Administration of Traditional Chinese Medicine. ^39^ Systematic reviews and meta-analyses have indicated the promising therapeutic effects of acupuncture on treating depression and anxiety. The effect sizes were estimated to be Hedges’ g of 0.41 (95% CI 0.18 to 0.63; p<0.001) for depression ^40^ and a standard mean effect size of 0.41(95% CI 0.31 to 0.50; p<0.001) for anxiety, ^41^ which both were of small to moderate magnitude. ^42^ These findings are consistent with earlier systematic reviews that included 12 randomized controlled trials showing that acupuncture is beneficial for treating anxiety disorders and perioperative anxiety, especially in auricular acupuncture. ^43^ Crucially, network meta-analyses suggest that electroacupuncture is as effective as CBT in alleviating depressive symptoms in subthreshold depression. ^44^ However, acupuncture’s key advantage lies in its scalability. Even with similar session requirements, ^45^^,^^46^ acupuncture benefits from a larger workforce and the availability of efficient models like community acupuncture, where one practitioner can treat multiple patients simultaneously. In contrast, CBT relies on a limited workforce of therapists for either individual or intensive group facilitation.

Chinese herbal formula such as Xiao Yao San (Free Wanderer Powder), Chai Hu Shu Gan San (Bupleurum Liver-Soothing Powder), and Gan Mai Da Zao Tang (Licorice, Wheat and Jujube Decoction) have also been demonstrated to elicit comparable efficacy as antidepressants in reducing the depression severity measured by Hamilton Depression Rating Scale (HDRS). ^47^ In a randomized controlled trial (RCT), Lycium barbarum polysaccharide, an active extract derived from the herbal medicine Goji berries, significantly reduced depressive symptoms compared to placebo in patients with subthreshold depression, demonstrating a large effect size (Cohen’s d = 0.86, p = 0.014). ^48^ Furthermore, the Chinese herbal medicine has been reported to be associated with fewer adverse events compared to psychotropic medications. A meta-analysis revealed that subjects taking herbal medicine were less likely to report adverse events than those taking antidepressants (pooled rate ratio (RAR) = 0.23, 95% CI: 0.16 to 0.33, p < 0.00001, I ^2^ = 59%). Additionally, the combination of Chinese herbal medicine and antidepressants was associated with fewer adverse events compared to antidepressants alone (pooled RAR = 0.43, 95% CI: 0.35 to 0.52, p < 0.00001, I ^2^ = 64%) in the treatment of depression. ^47^ Another meta-analysis indicated that the incidence of adverse events in the herbal formula Xiao Yao San group was lower than in the anxiolytics group, and the rates of adverse events in the group combining Xiao Yao San with anxiolytics were significantly lower than in the anxiolytics-only group. ^49^ Given this evidence of efficacy and safety, leveraging established, evidence-based formulas is recommended for treatment in primary care.

Beyond acupuncture and herbal therapies, other CM modalities also demonstrate robust therapeutic potential. For instance, acupressure has been shown to significantly reduce anxiety (a reduction in standardized mean difference (SMD) of 1.152, 95% CI 0.847 to 1.459; p<0.001), particularly effective in providing immediate relief for pretreatment anxiety. ^50^ CM-based integrated health interventions had larger effects on reducing depressive symptoms (SMD = −2.05, 95% CI: −2.74 to −1.37; p < 0.00001) compared with usual care, and showed no significant differences in reducing depression symptoms compared to CBT. ^51^ Similarly, mind-body exercises rooted in CM, such as Tai Chi, have outperformed non-mindful exercises in improving anxiety (Hedges’s d = 0.28, 95% CI, 0.08 to 0.48, p = 0.008), depression (Hedges’s d = 0.20, 95% CI, 0.04 to 0.36, p = 0.018), and general mental health (Hedges’s d = 0.40, 95% CI, 0.08 to 0.73, p = 0.017) with small-to-moderate effect sizes. ^52^ Reviewing these aspects, CM offers promising potential for managing and alleviating a variety of symptoms associated with SMDs.

## 3. Benefits of Chinese Medicine as a First-line Subthreshold Mental Disorders Treatment in Primary Care

The Lancet Commission report suggests that task-shifting to non-specialist health workers can be an effective strategy to improve the availability of interventions in mental health care. ^18^ Integrating CM as one of the first-line treatments in primary care for SMDs aligns with these objectives and offers several additional benefits. Firstly, in terms of workforce availability and accessibility, East Asian regions such as mainland China and Hong Kong present a compelling model for integration. The National Administration of Traditional Chinese Medicine reports that there are over 1.5 million licensed CM practitioners already embedded in primary care in China, with an annual growth rate of 6.6% since 2015. ^53^ This growth rate surpasses that of primary health-care physicians in all other specialties combined over the past decade in China. ^54^ Hong Kong boasts a well-regulated CM industry, with over 10,000 CM practitioners significantly contributing to primary care. ^55^ This infrastructure enables CM to immediately address workforce shortages in resource-limited areas with established CM systems but inadequate mental health staffing. ^56^

Second, cultural congruence and reduced stigma play a pivotal role in help-seeking behavior. Psychiatric labels often trigger self-stigma, deterring individuals from seeking conventional mental health care. ^57^ In contrast, CM employs a holistic framework that conceptualizes mental and physical health as interconnected aspects of overall well-being. ^58^ By emphasizing balance restoration, preventive care, and symptom management without pathological labeling, ^59^ CM is perceived as a form of “health maintenance” rather than “mental illness treatment.” ^60^ Research has demonstrated that Chinese Americans perceive greater community attitudes of shame when accessing Western psychiatric services as opposed to CM for treating mental disorders. ^61^ The medical paradigm of CM reduces barriers to care and fosters earlier intervention.

**Figure 1.**
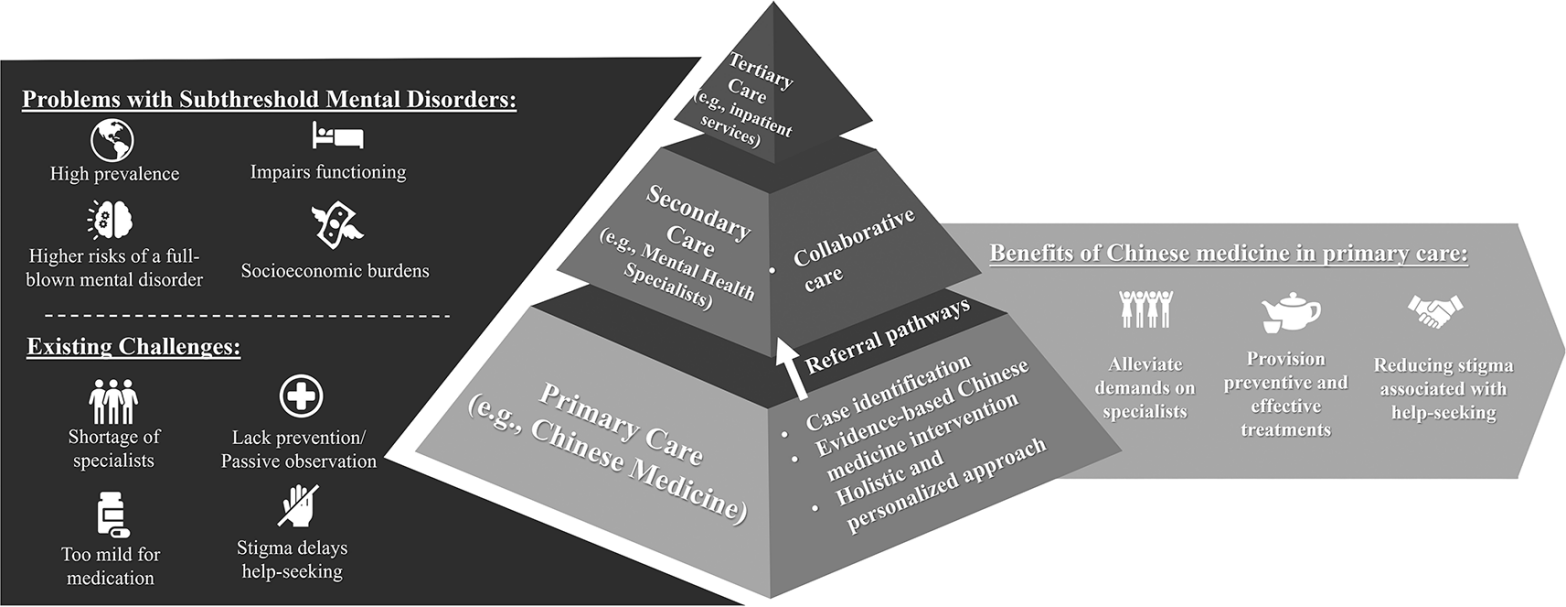
Integrating Chinese medicine in primary care to address subthreshold mental disorders challenges.

Third, integrating CM offers a compelling economic advantage. In mainland China, basic public health insurance schemes have progressively expanded their coverage to include CM treatments. ^62^ Similarly, the government of Hong Kong subsidizes care in its 18 territory-wide Chinese Medicine Clinics for Training and Research, providing a clear precedent for publicly supported services that could be scaled to include SMD management. ^63^ Beyond funding, CM demonstrates strong potential for cost-effectiveness compared to standard interventions. For instance, a trial found that while acupuncture and counseling for depression yielded similar health gains as measured in Quality-Adjusted Life Years (QALYs), acupuncture incurred significantly lower total costs to the health system (£1,227 vs. £1,450) over 12 months. ^64^ The Incremental Cost-Effectiveness Ratio (ICER) further underscored this, indicating that adopting counseling over acupuncture would cost the health service over £71,000 for each additional QALY gain, a figure exceeding standard thresholds for value in healthcare. ^64^ By integrating CM into first-line primary stepped-care models, health systems can provide earlier, cost-effective, and less stigmatized interventions while preserving specialist resources for higher-acuity needs ( Figure 1).

## 4. Challenges and Solutions in Integrating Chinese Medicine into Primary Care

Even in regions where CM is well-established, integrating it into primary care for mental health faces three significant challenges. First, there is a public lack of recognition regarding the role of CM in treating mental disorders. For instance, despite CM’s prevalence in Hong Kong, a territory-wide psychiatric epidemiological study in Hong Kong showed that a mere 1.8% of patients dealing with mental health issues would seek help from CM. ^65^ This is four times less than the proportion of patients seeking help for other health conditions, highlighting a significant underutilization and a lack of perceived legitimacy in the context of mental health. ^65^ Second, there is a lack of collaboration and defined referral pathways between CM and mental health specialties in secondary care. ^66^ The absence of clear, standardized procedures to guide CM practitioners and conventional healthcare providers in referring patients to each other can lead to disjointed and ineffective care. This deficiency often leads to delays in accessing advanced treatments for treatment-resistant patients, inadequate management of comorbid conditions, and insufficient specialist assessments or diagnoses needed for legal compliance. Such inefficiencies not only obstruct the integration of CM into mainstream mental health care but also potentially place CM at a disadvantage within the primary care setting. Third, owing to different theological and cultural backgrounds and knowledge gaps, other healthcare providers in the mental health field often have varying perceptions of CM’s efficacy, which can hinder its integration. ^67^ Addressing these challenges requires a multi-pronged approach. Public education campaigns can raise awareness about the potential benefits of CM in treating mental disorders, presenting scientific evidence to enhance its perceived legitimacy. Concurrently, research should be encouraged to further explore and validate the efficacy of CM treatment in this field. Furthermore, the development of collaborative frameworks and referral pathways is essential to facilitate collaboration between CM and other mental health specialties. This could be realized through policy changes and the formulation of clear collaboration and referral guidelines. Lastly, cross-disciplinary education can play a pivotal role in improving understanding of CM among other mental health practitioners. By embedding CM into medical curricula, conducting joint training programs, and promoting regular dialogues and interdisciplinary exchanges, practitioners’ knowledge and acceptance of CM can be improved, thus paving the way for its successful integration into primary mental healthcare.

## 5. Conclusion

In conclusion, SMDs represent a significant public health burden, and current management strategies are often hindered by workforce shortages and stigma. This perspective has argued that CM emerges as a promising first-line option, particularly in primary care settings with established infrastructure and public acceptance. With its holistic approach, growing evidence of efficacy, and potential to be a less-stigmatized alternative, integrating CM in these systems could enhance help-seeking and alleviate workforce gaps. The successful integration of CM in these model regions could provide a valuable, evidence-based blueprint for other healthcare systems to consider in the future. To realize this potential, a focused strategy encompassing public education, rigorous research, policy reform to create clear referral pathways, and interdisciplinary training is essential.

## Ethical approval

Ethical approval and consent were not required.

## Declaration of generative AI and AI-assisted technologies in the writing process

The authors declare that they have not used generative AI and AI-assisted technologies in the writing process.

## Data Availability

No data associated with this article.
