# Supplementary material for: Chinese Medicine as First-Line Treatment for Subthreshold Mental Disorders in Primary Care: Opportunities and Challenges
Source: F1000Res. 2025 Oct 9;14:475. Originally published 2026 May 6. [Version 2] doi: 10.12688/f1000research.163621.2 (PMC13228993; doi:10.12688/f1000research.163621.2)
Supplement: Peer review history

# Reviewer report: Reviewer response for version 2

**DOI:** 10.5256/f1000research.189428.r473351

**Published:** 2026-04-21

**Authors:** Yujin Choi

**Affiliations:** 1 Korea Institute of Oriental Medicine, Daejeon, South Korea

This revised manuscript presents a well-structured and focused argument for integrating Chinese medicine as a first-line treatment for subthreshold mental disorders in primary care settings. I have the following minor comments for the authors' consideration.

1. The abbreviation "SMD" is used in this manuscript to denote both "subthreshold mental disorders" (as a population category) and "standardized mean difference" (as a statistical effect size measure). These dual uses appear in close proximity throughout the text and may cause confusion for readers. The authors should consider differentiating these.
2. Several effect sizes cited in Section 2 to support the efficacy of CM interventions are drawn from studies enrolling patients with diagnosed mental disorders or other clinical populations (e.g., perioperative anxiety), rather than individuals with subthreshold presentations specifically. Given that the manuscript's central argument concerns SMDs, a brief clarification of the extent to which these estimates can be extrapolated to subthreshold populations is required.

Is the topic of the opinion article discussed accurately in the context of the current literature?

Yes

Are arguments sufficiently supported by evidence from the published literature?

Yes

Are all factual statements correct and adequately supported by citations?

Yes

Are the conclusions drawn balanced and justified on the basis of the presented arguments?

Yes

Reviewer Expertise:

Integrative medicine, Korean medicine, Neuropsychiatry

I confirm that I have read this submission and believe that I have an appropriate level of expertise to confirm that it is of an acceptable scientific standard.

# Reviewer report: Reviewer response for version 1

**DOI:** 10.5256/f1000research.180003.r411863

**Published:** 2025-09-17

**Authors:** Li Deng

**Affiliations:** 1 Jinan University, Guangzhou, Guangzhou, China

This perspective article presents a forward-looking and practically significant proposition: utilizing Chinese Medicine (CM) as a first-line treatment option for Subthreshold Mental Disorders (SMDs) within primary care. With a clear structure, the article systematically elaborates on the disease burden of SMDs, current treatment challenges, the potential efficacy and advantages of CM, as well as the obstacles and solutions related to its integration.

 As Reviewer 1 pointed out, the overly broad scope of the research constitutes a significant limitation. In most Western developed countries, challenges such as the lack of unified practice standards and regulatory frameworks, disparities between efficacy evaluation systems and biomedical models, potential skepticism among Western medical practitioners, and the high costs associated with cultural translation and patient education remain unresolved. These issues render the proposed integration strategies abstract and lacking in operational practicality, making it difficult to translate them into concrete practice guidelines across different contexts.

 Although the authors mention that Chinese medicine may be "cost-effective" and cite a limited number of studies (such as one acupuncture trial), this argument lacks sufficient depth and persuasiveness. More rigorous pharmacoeconomic evidence is needed. This entails conducting strict cost-effectiveness analyses (CEA) or cost-utility analyses (CUA) that directly compare Chinese medicine interventions with currently recommended first-line treatment options—such as cognitive behavioral therapy (CBT), mindfulness-based interventions (MBIs), or active monitoring. The key is not only to demonstrate the efficacy of Chinese medicine but also to prove that it can reduce overall healthcare costs or societal resource burdens while achieving equivalent or better health outcomes. At present, such high-quality economic evaluations remain relatively scarce in the field of Chinese medicine. If the article were to adequately address this gap, its value would be significantly enhanced.

 While the authors list various Chinese medicine therapies (e.g., acupuncture, specific herbal formulas, tai chi, etc.) and summarize evidence of their efficacy, they fail to elaborate in depth on how to standardize and scale these applications in primary care settings. Furthermore, a critical question remains unaddressed: Should standardized fixed formulas, which facilitate quality control, RCT execution be recommended, or should traditional pattern-based diagnosis and treatment—which allows for individualized care aligned with Chinese medicine theory but introduces complexity and challenges to standardization—be permitted? The article does not engage with this essential decision-making dilemma.

Is the topic of the opinion article discussed accurately in the context of the current literature?

Yes

Are arguments sufficiently supported by evidence from the published literature?

Yes

Are all factual statements correct and adequately supported by citations?

Yes

Are the conclusions drawn balanced and justified on the basis of the presented arguments?

Yes

Reviewer Expertise:

The article is already ​largely comprehensive​ in its current state.

I confirm that I have read this submission and believe that I have an appropriate level of expertise to confirm that it is of an acceptable scientific standard.

# Reviewer report: Reviewer response for version 1

**DOI:** 10.5256/f1000research.180003.r383877

**Published:** 2025-05-27

**Authors:** Rachael Frost

**Affiliations:** 1 Liverpool John Moores University, Liverpool, England, UK

This opinion article advocates for the inclusion of Chinese Medicine as a first line treatment for SMDs. It demonstrates an interesting and generally well supported argument for greater inclusion of CM, and highlights relevant potential benefits and challenges. I feel it would benefit from considering the following:

 1) A narrower focus on which countries the authors are recommending integration in, as there are many different systems and consequent issues globally. It seems integration should first be recommended in China as somewhere with existing infrastructure and high CM acceptance/interest? The countries should be clear as it has implications for how integration would happen - for example in Western countries there would be a greater challenge of integrating private services into publicly funded care. Alleviating workforce gaps would be good but this is only likely to occur in specific Asian countries where there are high rates of well trained CM practitioners - they may also face a shortage in other countries!

 2) More economic consideration is needed. Who are the authors proposing should pay for the integration? There is some economic evidence cited but it would be good to expand this, particularly in comparison to existing interventions.

 3) It would be good to comment on the comparability in terms of delivery of acupuncture vs CBT with regards to  N sessions needed per person for effects, given that the effect sizes are very similar but different session duration or number has workforce and delivery implications. For this reason antidepressants are often given instead of CBT. Are the authors proposing CM herbal mixes be individually tailored or using established products only?

 4) Section 2 would benefit from being split into 2 paragraphs to enhance readability. 

 5) Statement "acupressure has been shown to significantly reduce anxiety (standardized mean difference (SMD) = 1.152, 95% CI 0.847 to 1.459; p<0.001)" - number is positive, implying an increase? It ma be worth restating as "a reduction of 1.152" for clarity.

Is the topic of the opinion article discussed accurately in the context of the current literature?

Yes

Are arguments sufficiently supported by evidence from the published literature?

Partly

Are all factual statements correct and adequately supported by citations?

Yes

Are the conclusions drawn balanced and justified on the basis of the presented arguments?

Yes

Reviewer Expertise:

Western herbal medicine, primary care, mental health, depression, anxiety

I confirm that I have read this submission and believe that I have an appropriate level of expertise to confirm that it is of an acceptable scientific standard, however I have significant reservations, as outlined above.

# Response

**Published:** 2025-10-07

**Authors:** Hiu To Tang

**Affiliations:** Hong Kong Baptist University, Hong Kong, Hong Kong

Dear Dr. Frost,

 "Chinese Medicine as First-Line Treatment for Subthreshold Mental Disorders in Primary Care: Opportunities and Challenges"

 Thank you for your time and the thorough reading of our manuscript. We are grateful for the insightful and favourable comments. We have revised the manuscript taking into consideration all the feedback received. For clarity, the reviewers’ comments are presented in bold, our responses are in regular font, and the corresponding changes in the manuscript are in italic. We believe these revisions have significantly strengthened the manuscript's argument. We hope we have successfully addressed all the concerns and that the revised manuscript is now suitable for publication in F1000Research. Thank you for your time and consideration.

 Yours sincerely,

 Tang Hiu To

**1) A narrower focus on which countries the authors are recommending integration in, as there are many different systems and consequent issues globally. It seems integration should first be recommended in China as somewhere with existing infrastructure and high CM acceptance/interest? The countries should be clear as it has implications for how integration would happen - for example in Western countries there would be a greater challenge of integrating private services into publicly funded care. Alleviating workforce gaps would be good but this is only likely to occur in specific Asian countries where there are high rates of well trained CM practitioners - they may also face a shortage in other countries!**

**Response:** Thank you for this insightful comment. We agree that a global recommendation is too broad and overlooks critical systemic differences. Accordingly, we have substantially revised the manuscript to narrow our focus to regions where integration is most feasible due to existing infrastructure, public acceptance, and a robust workforce. Our recommendation now centers on East Asian regions, specifically using mainland China and Hong Kong as primary examples. This revised focus allows for a more nuanced discussion of the implementation model and its potential to alleviate workforce gaps in these specific contexts. We have also added a brief discussion acknowledging the distinct challenges that integration would face in other healthcare systems.

**Revise** **d manuscript: **

- ***Abstract:** This perspective advocates for the integration of Chinese medicine (CM) as a first-line treatment for SMDs, focusing specifically on primary care settings in regions with established CM infrastructure and high public acceptance.*
- ***Introduction:** In confronting these challenges, Chinese medicine (CM) presents as one of the promising first-line treatment options in primary care for the management of SMDs, particularly within primary care systems that have existing infrastructure and high public acceptance, such as those in mainland China and Hong Kong.*
- *Similarly, 10% of the Chinese population exhibits symptoms of a subthreshold anxiety disorder, significantly higher than the 3.5% who fulfill the criteria for a clinically diagnosed anxiety disorder. *
- ***Benefits of Chinese Medicine as a First-line Subthreshold Mental Disorders Treatment in Primary Care:** Integrating CM as one of the first-line treatments in primary care for SMDs aligns with these objectives and offers several additional benefits. Firstly, in terms of workforce availability and accessibility, East Asian regions such as mainland China and Hong Kong present a compelling model for integration. The National Administration of Traditional Chinese Medicine reports that there are over 1.5 million licensed CM practitioners already embedded in primary care in China, with an annual growth rate of 6.6% since 2015. ^53^ This growth rate surpasses that of primary health-care physicians in all other specialties combined over the past decade in China. ^54^ Hong Kong boasts a well-regulated CM industry, with over 10,000 CM practitioners significantly contributing to primary care. ^55^ This infrastructure enables CM to immediately address workforce shortages in resource-limited areas with established CM systems but inadequate mental health staffing. ^56^*
- ***Challenges and Solutions in Integrating Chinese Medicine into Primary Care** **:** Even in regions where CM is well-established, integrating it into primary care for mental health faces three significant challenges.*
- *For instance, despite CM's prevalence in Hong Kong, a territory-wide psychiatric epidemiological study in Hong Kong showed that a mere 1.8% of patients dealing with mental health issues would seek help from CM. ^65^*
- ***Conclusion:** In conclusion, SMDs represent a significant public health burden, and current management strategies are often hindered by workforce shortages and stigma. This perspective has argued that CM emerges as a promising first-line option, particularly in primary care settings with established infrastructure and public acceptance. With its holistic approach, growing evidence of efficacy, and potential to be a less-stigmatized alternative, integrating CM in these systems could enhance help-seeking and alleviate workforce gaps. The successful integration of CM in these model regions could provide a valuable, evidence-based blueprint for other healthcare systems to consider in the future. To realize this potential, a focused strategy encompassing public education, rigorous research, policy reform to create clear referral pathways, and interdisciplinary training is essential.*

**2) More economic consideration is needed. Who are the authors proposing should pay for the integration? There is some economic evidence cited but it would be good to expand this, particularly in comparison to existing interventions.**

**Response:** Thank you for this crucial feedback. We agree that a discussion on funding models and a more direct comparative economic analysis is essential to the manuscript's argument. We have now explicitly identified existing public funding pathways in the proposed model regions and strengthened the cost-effectiveness evidence by including a direct comparison from a key clinical trial, comparing non-pharmacological treatments- acupuncture and counseling.

**Revise** **d manuscript:**

- ***Benefits of Chinese Medicine as a First-line Subthreshold Mental Disorders Treatment in Primary Care: **Third, integrating CM offers a compelling economic advantage. In mainland China, basic public health insurance schemes have progressively expanded their coverage to include CM treatments. ^62^ Similarly, the government of Hong Kong subsidizes care in its 18 territory-wide Chinese Medicine Clinics for Training and Research, providing a clear precedent for publicly supported services that could be scaled to include SMD management. ^63^ Beyond funding, CM demonstrates strong potential for cost-effectiveness compared to standard interventions. For instance, a trial found that while acupuncture and counseling for depression yielded similar health gains as measured in Quality-Adjusted Life Years (QALYs), acupuncture incurred significantly lower total costs to the health system (£1,227 vs. £1,450) over 12 months. ^64^ The Incremental Cost-Effectiveness Ratio (ICER) further underscored this, indicating that adopting counseling over acupuncture would cost the health service over £71,000 for each additional QALY gain, a figure exceeding standard thresholds for value in healthcare. ^64^ By integrating CM into first-line primary stepped-care models, health systems can provide earlier, cost-effective, and less stigmatized interventions while preserving specialist resources for higher-acuity needs( Figure 1).*

**3) It would be good to comment on the comparability in terms of delivery of acupuncture vs CBT with regards to  N sessions needed per person for effects, given that the effect sizes are very similar but different session duration or number has workforce and delivery implications. For this reason antidepressants are often given instead of CBT. Are the authors proposing CM herbal mixes be individually tailored or using established products only?**

**Response:** Thank you for your insightful feedback. You raised an important point regarding the practical delivery of acupuncture versus CBT, particularly concerning session numbers and workforce implications. Addressing this point strengthens our argument for integrating CM. While the effect sizes are similar, the delivery models have distinct workforce implications. A typical course of CBT involves 8–16 weekly sessions of approximately 50 minutes each, requiring a highly trained therapist (Cuijpers et al., 2013). Acupuncture protocols for depression or anxiety often involve a similar number of sessions, such as 10–12 sessions over 8–12 weeks, with each session lasting 30–60 minutes (MacPherson, 2013). However, the larger and more rapidly growing workforce of licensed CM practitioners makes acupuncture a more scalable intervention in settings like mainland China and Hong Kong. Furthermore, models like community acupuncture, where one practitioner can treat multiple patients in a shared space, offer a highly efficient delivery method that can alleviate workforce pressures—a significant advantage over the one-on-one format or intensive group facilitation required for CBT. To clarify our proposed approach to CHM, we now specify that our model prioritizes established, evidence-based formulas for first-line treatment in primary care. This approach is crucial for ensuring the safety, scalability, and standardization required for broad implementation. Accordingly, we have added the following text in Section 2:

**Revised manuscript: **

- ***Chinese Medicine and Subthreshold Mental Disorders:*** *Crucially, network meta-analyses suggest that electroacupuncture is as effective as CBT in alleviating depressive symptoms in subthreshold depression. ^44 ^However, acupuncture's key advantage lies in its scalability. Even with similar session requirements, ^45,46 ^acupuncture benefits from a larger workforce and the availability of efficient models like community acupuncture, where one practitioner can treat multiple patients simultaneously. In contrast, CBT relies on a limited workforce of therapists for either individual or intensive group facilitation. * 
- *Given this evidence of efficacy and safety, leveraging established, evidence-based formulas is recommended for treatment in primary care. * 

**References**

 Cuijpers, P., Berking, M., Andersson, G., Quigley, L., Kleiboer, A., & Dobson, K. S. (2013). A meta-analysis of cognitive-behavioural therapy for adult depression, alone and in comparison with other treatments. The Canadian Journal of Psychiatry, 58(7), 376-385.

 MacPherson, H., Richmond, S., Bland, M., Brealey, S., Gabe, R., Hopton, A., ... & Watt, I. (2013). Acupuncture and counselling for depression in primary care: a randomised controlled trial. PLoS medicine, 10(9), e1001518.

**4) Section 2 would benefit from being split into 2 paragraphs to enhance readability.**

**Response: **Thank you for this suggestion to improve readability. We agree and have restructured Section 2 accordingly, dividing it into four focused paragraphs. The new structure now separately covers the evidence for clinical guidelines, acupuncture, Chinese herbal medicine, and other CM modalities. We believe this provides a clearer and more logical progression of the evidence presented.

**5) Statement "acupressure has been shown to significantly reduce anxiety (standardized mean difference (SMD) = 1.152, 95% CI 0.847 to 1.459; p<0.001)" - number is positive, implying an increase? It ma be worth restating as "a reduction of 1.152" for clarity.**

**Response: **Thank you for this observation. To improve the clarity of this statement, we have revised the sentence in the manuscript.

- **Revised manuscript:** *For instance, acupressure has been shown to significantly reduce anxiety (a reduction in standardized mean difference (SMD) of = 1.152, 95% CI 0.847 to 1.459; p<0.001), particularly effective in providing immediate relief for pretreatment anxiety.* ^50^
